# Multi-Kinase Inhibition by New Quinazoline–Isatin Hybrids: Design, Synthesis, Biological Evaluation and Mechanistic Studies

**DOI:** 10.3390/ph18101546

**Published:** 2025-10-14

**Authors:** Mohammed M. Alanazi, Reem I. Al-Wabli

**Affiliations:** Department of Pharmaceutical Chemistry, College of Pharmacy, King Saud University, Riyadh 11451, Saudi Arabia

**Keywords:** quinazoline, isatin, molecular hybridization, antitumor activity

## Abstract

**Background/Objectives:** Cancer is a worldwide health concern and is the second leading cause of death, responsible for nearly one in six deaths. Discovery of new anticancer agents is still a challenge for medicinal chemists and further research will improve patients’ chances of survival. Protein kinases are among the most popular and successful biological targets for developing anticancer drugs. In this context, protein kinases were selected as targets, and a series of isatin–quinazoline hybrids were synthesized. **Methods:** Their antiproliferative activity was evaluated against four cancer cell lines (HepG2, MCF-7, MDA-MB-231, and HeLa) and one normal fibroblast cell line (WI38) using MTT assays. **Results:** The tested compounds showed variable cytotoxic effects on the four cancer cell lines. Compound **6c** exhibited the most potent anticancer activity against all cancer cells. In addition, this compound was tested for the effect on the expression of anti-apoptotic Bcl-2 protein and pro-apoptotic proteins Bax, caspase-3, and caspase-9, which revealed induction of apoptosis similar to staurosporine. Furthermore, an annexin V-FITC/PI dual staining assay confirmed that compound **6c** induced cell death by apoptosis. Flow cytometric analysis revealed that compound **6c** induced cell cycle arrest at the sub-G1 and S phases in the HepG2 cell line. Moreover, compound **6c** was found to be a multi-kinase inhibitor with potent inhibitory activity on CDK2, EGFR, VEGFR-2, and HER2, with IC_50_ values of 0.183 ± 0.01, 0.083 ± 0.005, 0.076 ± 0.004, and 0.138 ± 0.07 μM, respectively. Finally, a molecular docking simulation was conducted to predict possible binding interactions with the active site of CDK2. **Conclusions:** These findings suggest that compound **6c** is a promising multi-kinase inhibitor with potent anticancer activity, warranting further investigation as a potential therapeutic agent.

## 1. Introduction

According to World Health Organization (WHO) reports, cancer is a huge group of diseases characterized by abnormal cell growth that can affect all organs and tissues of the body. The latter process, called metastasis, is the leading cause of death from cancer. Cancer is also referred to as neoplasm or malignant tumor [1]. Mutations cause alteration in cellular DNA leading to genetic instability that change the ordinary cellular functions. In 2011, a paper published by Hanahan and Weinberg supported the continued use of the definition of the “hallmark of cancer” as the primary biological events that develop throughout tumor formation [2,3]. Hallmarks of cancer cells compared to normal cells include sustaining growth signaling, escaping growth suppressors, resisting apoptosis, allowing replicative immortality, stimulating angiogenesis, enabling invasion and metastasis, the capability to modify or reprogram cellular metabolism, avoiding immune destruction, genome instability and mutation, and tumor-promoting inflammation [2,4]. Numerous therapeutic targets have been studied, such as alkylating agents like chlorambucil and nitrogen mustard, which are used to treat Hodgkin’s disease; pyrimidines and tubulin-binding medications like the vinca alkaloids that disrupt tubulin function; antimetabolites that mimic naturally occurring purines; and organoplatinum complexes like cisplatin and carboplatin, which have been in use since the 1970s [5,6]. Chemotherapy has a number of drawbacks, such as numerous adverse effects brought on by the cytotoxic nature of the medications and their low selectivity. Drug resistance, however, is a major drawback that can arise during treatment and reduce efficacy [7,8,9]. Protein kinases are among the most widely used and effective biological targets for anticancer medications [10]. Kinases are catalytic proteins that have the ability to move a phosphoryl group (PO_3_^2−^) from ATP to their substrates, which may be proteins, lipids, or carbohydrates [11]. It is well known that the phosphorylation reaction is very substrate-specific; in the case of protein kinases, the phosphorylatable residue (a tyrosine, serine, or threonine) should be surrounded by an amino acid sequence in the protein substrate [12]. This transfer then changes the folding of the substrate and triggers or inhibits its specific function [13]. Kinases are primarily inactive, although phosphorylation (either by another kinase or by themselves, subsequently referred to as autophosphorylation) or binding to a ligand molecule, scaffold protein, or another kinase domain of the same kind can alter their activity level [14]. Lastly, the conformation-altering effect of kinases is primarily transient because phosphatase enzymes counteract their action by continuously dephosphorylating the substrate molecules [15]. Kinase activity is shared by protein kinases through a conserved structural module known as a domain. This domain consists of two lobes: the amino-terminal lobe and the carboxy-terminal lobe. The Mg ATP-binding site in the center of the protein is formed by the flexible hinge region connecting these two lobes [14]. A kinase can be a substrate for another kinase forming a kinase cascade, a common type of signaling pathway. Signaling pathways mean signal transduction from plasma membrane receptors to transcription factors inside the nucleus. Ultimately, transcription factors regulate the transcription of genes, and the resulting proteins influence various cellular functions: transcription of genes and metabolism, growth, division, motility, or apoptosis of cells [16].

Protein kinases have become the most popular pharmacological targets in the past 20 years because increases in their activity are common in a number of diseases, including diabetes, cardiovascular, neurological, and inflammatory conditions, and cancer. Inhibiting these kinases has been shown to reduce some of the symptoms [17]. Given the significant role of kinases in signaling pathways and biological functions, it is not uncommon that several of these are involved in cancer [18]. In normal cells, the amount of cellular tyrosine kinase phosphorylation is strongly controlled [16]. Kinases can be involved in cancer in several ways, including misregulated expression and/or amplification, aberrant phosphorylation, mutation, chromosomal translocation, and epigenetic regulation [19]. Inhibiting kinase activity in normal cells can be tolerated, allowing selective targeting of tumor cells. Despite this fact, developing selective kinase inhibitors can be difficult because of the high level of conservation, particularly at the ATP binding site, among the different kinases [20]. Attempts to overcome resistance to some of the marketed anticancer drugs led to an vital need to come up with new methods to fight this disease, and the concept of combination chemotherapy was introduced [6,7]. Some disadvantages associated with combination chemotherapy include patient non-compliance due to increased dosing frequency and forgetting to take medication [21]. In molecular hybridization strategies, two or more drugs are chemically combined in one chemical structure to solve complications of combination chemotherapy caused by patient non-compliance [22]. Molecular hybridization is a strategy for rational designing of new ligands with combinations of two or more known pharmacophoric moieties, resulting in a new hybrid retaining preselected characteristics of the original moieties [23]. The choice of drugs or pharmacophores is typically guided by their known pharmacological activities, aiming to identify novel chemical entities with high potency. Hybrid molecules are anticipated to demonstrate distinct and/or dual mechanisms of action, altered selectivity profiles, and potentially fewer side effects [24]. In fact, the position and nature of substitutions are very crucial for determining the biological activity of different quinazoline derivative [25]. According to a review published by Auti and coauthors, quinazoline or isatin hybrids have been successfully synthesized and several compounds have shown good anticancer activity [26]. Furthermore, Fares and coworkers have reported the synthesis of several quinazoline–isatin hybrids connected through hydrazine. Some of the synthesized compounds revealed potent anticancer activity with apoptosis-inducing effects [27]. Finally, a survey of isatin hybrids and their biological properties was investigated which highlighted the anticancer effect of isatin [28]. Encouraged by FDA-approved quinazoline and isatin-containing anticancer drugs [23,29,30,31], a compound containing both moieties in one single molecule can result in the following advantages: enhanced affinity, efficacy, and a selectivity profile with reduced undesirable adverse effects compared to the parent compounds [32,33]. The hybridization approach has already been applied to develop novel anticancer agents that displayed potent nM activities [34]. Therefore, hybrid anticancer compounds containing quinazoline and isatin were designed, synthesized, and evaluated for anticancer activity.

## 2. Results and Discussion

### 2.1. Chemistry

**Scheme 1:**
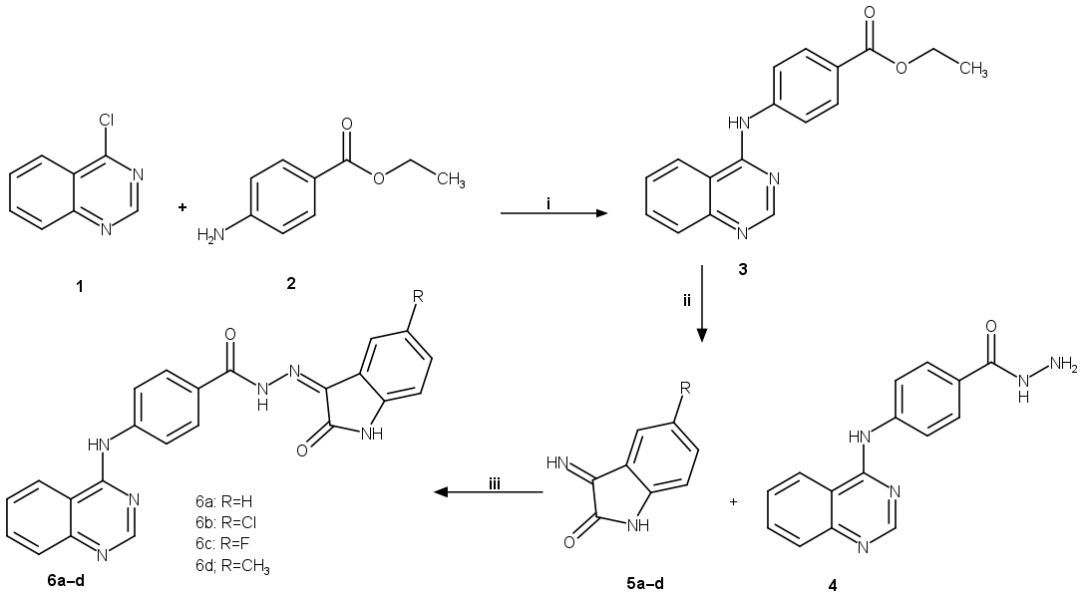
Synthesis of the target hybrid compounds, **6a**–**d**. Reagents and conditions: (i) absolute ethanol/reflux 6 h; (ii) absolute ethanol and N_2_H_4_-H_2_O/reflux 8 h; (iii) absolute ethanol and AcOH/reflux 8 h.

The general synthetic procedure for the synthesis of the hybrid compounds is demonstrated in Scheme 1. The initial step resulted in the formation of ethyl 4-[(quinazolin-4-yl)amino]benzoate (**3**) by refluxing 4-chloroquinazoline (**1**) in absolute ethanol and ethyl 4-aminobenzoate (**2**) for 6 h. Afterwards, subsequent reflux of intermediate **3** with hydrazine hydrate 80% yielded 4-[(quinazolin-4-yl)amino]benzohydrazide (**4**). Finally, intermediate 4 was treated with either istatin, 5-flouroisatin, 5-chloroisatin, or 5-methylisatin (**5a**–**d**) to obtain *N*′-(2-oxoindolin-3-ylidene)-4-(quinazolin-4-ylamino)benzohydrazide or *N*′-(5-subsitituted-2-oxoindolin-3-ylidene)-4-(quinazolin-4-ylamino)benzohydrazide derivatives (**6a**–**d**). The reaction was performed by refluxing absolute ethanol treated with 0.2 mL of glacial acetic acid for 8 h. The structures of the compounds were assessed via elemental analysis and spectral data, including mass spectrometry (MS), and proton and carbon nuclear magnetic resonance spectroscopy (^1^H NMR and ^13^C NMR). Physical properties including color and melting points were reported. The presence of multiple signals in the ^1^H NMR spectra between 7 and 9 ppm indicated the existence of aromatic protons in compounds **6a**–**d**. Moreover, the spectra of the synthesized compounds showed a D_2_O-exchangeable signal of a NH proton of the isatin moiety in the region 11.20–11.50 ppm. A distinctive signal of the Schiff base N=CH group appeared as a singlet in the range of 8.7 to 8.8 ppm, for all synthesized compounds. Finally, mass spectra revealed molecular ion [M−H]^−^ peaks of different intensity.

### 2.2. Biological Study

#### 2.2.1. Cytotoxicity Assay/Anti-Proliferative Activity

The synthesized isatin–quinazoline compounds (**6a**–**d**) were assessed for their cytotoxicity against four cancer cell lines, namely, hepatocellular carcinoma HepG2, breast carcinoma MCF-7, breast adenocarcinoma MDA-MB-231, and cervical carcinoma HeLa, using an MTT colorimetric assay [35]. Doxorubicin and sunitinib were used as positive controls for comparison. The antiproliferative activities are expressed as the median growth inhibitory concentration (IC_50_) in micromolar range and are described in Table 1.

From the results in Table 1, it is obvious that most of the synthesized compounds exhibited variable growth inhibitory activity against the tested cancer cell lines. Compound **6d** demonstrated weak inhibitory active against the four cell lines and surprisingly, it was more cytotoxic against the normal cell line (WI38). On the other hand, compound **6b** displayed moderate growth inhibitory activity with IC_50_ values of 61.35 ± 3.5, 70.65 ± 3.4, 56.31 ± 3.2, and 66.82 ± 3.4 µM against HepG2, MCF-7, MDA-MB-231, and HeLa, respectively. Furthermore, compounds **6a** and **6c** exhibited potent cytotoxic effects, with **6c** being more potent than the reference compounds sunitinib and doxorubicin against HepG2, MCF-7, MDA-MB-231 cell lines, with a comparable effect on HeLa cells. To investigate the selectivity of the latter three compounds, their cytotoxicity against WI38 was tested. Testing revealed a significant selectivity comparable to that of the tyrosine kinase inhibitor sunitinib and better than that of the cytotoxic agent doxorubicin, which did not discriminate between normal and cancer cells. These results showed that the antiproliferative activity of the synthesized compounds was favorable, in the order F > H > Cl > CH_3_. Fluorine is the smallest halogen and a strong electron-withdrawing group, therefore; it is likely that it enhances the antiproliferative activity by reducing the electron density of the isatin moiety, allowing better interaction with the target proteins. Moreover, its small size also minimizes steric hindrance, allowing better accommodation at the binding site. Hydrogen is also small in size but neutral in electronic effect; so, compound **6a** was less potent than compound **6c**. Conversely, chlorine is an electron-withdrawing group but it is bulker than fluorine and hydrogen, introducing steric hindrance that may interfere with proper binding. Finally, the electron-donating effect of methyl may disturb the binding interactions with target proteins and hence, compound **6d** showed a weak anticancer effect.

#### 2.2.2. Flow Cytometry Cell Cycle Analysis

**Figure 1:**
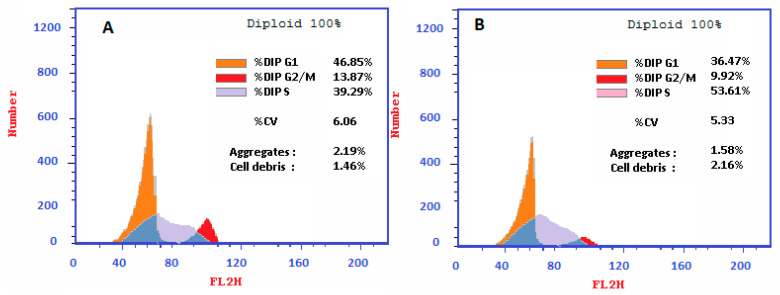
Representative histograms of DNA content distribution of cell cycle phases of HepG2 cell line. (**A**) Untreated cells. (**B**) Cells treated with compound **6c** for 24 h.

As compound **6c** showed the highest anti-proliferative activity against all tested cell lines, it was selected for further investigation of the mechanism of action. Its impacts on cell cycle progression and induction of apoptosis were investigated. The effect of compound **6c** on the regulation of the cell cycle progression was studied using a Propidium Iodide Flow Cytometry Kit assay. Alterations in the cell cycle were recorded after the treatment of HepG2 cells with 2.6 μM of compounds **6c**, chosen for its excellent antiproliferative activities within 24 h. As presented in Figure 1 and Table 2, compound **6c** induced a markable increase in the accumulation of cancer cells in the cell population in the sub-G1 population (42.79%) in comparison with the control (1.73%). Also, a higher cell accumulation (53.61%) was observed at the S phase for compound **6c** (control; 39.28%).

#### 2.2.3. Annexin V-FITC Dual-Staining Apoptosis Assay

**Figure 2:**
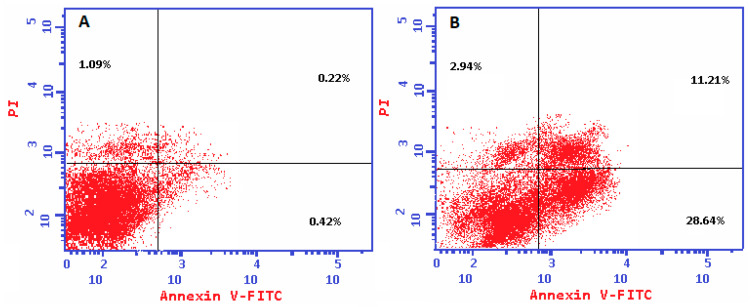
Dot plot of annexin V/PI-stained cells. (**A**) Untreated cells. (**B**) Cells treated with compound **6c** for 24 h.

Apoptosis can be detected by measuring translocated phosphatidylserine (PS) from the interior to the surface of the cell membrane [36]. Therefore, an annexin V-FITC/PI (fluorescein–isothiocyanate/propidium iodide) dual staining flow cytometric assay was carried out for compound **6c** to investigate the mechanism of cell death induced by the assessed compound. Analysis of early and late apoptosis showed the ability of compound **6c** to produce remarkable amounts of apoptosis within the respective exposed cells compared to the non-treated cells. The dot plot flow cytometry data of the cells stained with annexin V-FITC and PI is summarized in Table 3 and displayed in Figure 2.

#### 2.2.4. Gene Expression Analysis

One significant anticancer mechanism is apoptosis [37]. Drugs that induce apoptosis cause simultaneous or subsequent activation of death receptor systems, mitochondrial dysfunction, and caspase proteolytic processing [38]. Thus, the cell death pathway may occur in several locations. In this study, the alteration in Bax, Bcl-2, and caspase-3 and 9 gene expression following the stimulation of apoptosis by **6c** in HepG2 cells using the RT-qPCR method and staurosporine as a positive control is reported in Table 4.

The results show that there was upregulation in the proapoptotic protein, Bax, caspase-9 and caspase-3. Downregulation in antiapoptotic Bcl-2 protein was observed. These results along with the previous confirm that compound **6c** induced apoptosis of HepG2 cells.

#### 2.2.5. In Vitro CDK2, EGFR, VEGFR-2, and HER2 Inhibitory Assay

The synthesized compound **6c** and reference standards were evaluated for inhibitory effects against CDK2, EGFR, VEGFR-2, and HER2 protein kinases; the corresponding IC_50_ values are reported in Table 5. The results of this experiment showed that **6c** exhibit substantial inhibitory effects against all test protein kinases, with values of 0.083 μM (EGFR), 0.076 μM (VEGFR-2), 0.138 μM (HER2), and 0.183 (CDK2), indicating that compound **6c** exherts its anticancer effect and apoptotic cell death though multikinase inhibition effects.

### 2.3. In Silico Study

#### 2.3.1. Molecular Docking

The significant inhibition of CDK2 by compound **6c** prompted us to investigate the possible binding interactions of compound **6c** with the active site of CDK2 by performing molecular docking simulation using AutoDock Vina in PyRx 0.8.

**Figure 3:**
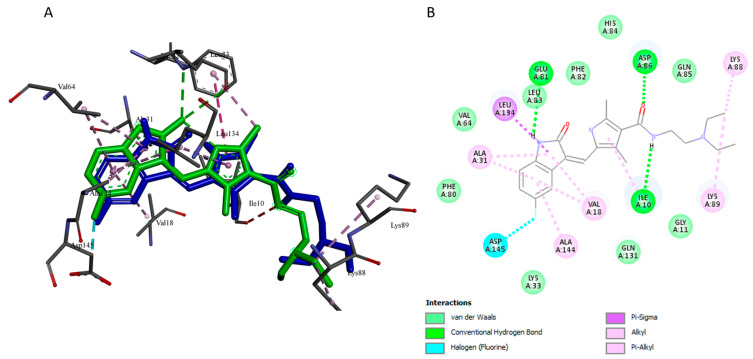
(**A**) Two-dimensional (2D) interactions of sunitinib redocked into the active site of CDK2 enzyme (PDB ID: 3TI1). (**B**) Three-dimensional (3D) interactions of the superimposition of the docking pose (blue) and the co-crystallized pose (green) of sunitinib.

The crystal structure of CDK2 in a complex with sunitinib (PDB code: 3TI1) was obtained from PDB. First, the co-crystallized ligand sunitinib was redocked into the CDK2 active site to validate the docking technique. The docking protocol’s suitability for the next docking investigation was verified by the redocking validation step. The highly observed superimposition between the docked pose and the co-crystallized inhibitor pose of 1.6 Å, as well as the docking score of −9 kcal/mol and the small root mean standard deviation (RMSD) between them, serves as evidence for this (Figure 3A). Sunitinib interacts with CDK2 with the amino acid Val18, Ala31, Leu83, Leu134, and Asp145. Furthermore, sunitinib was stabilized by hydrophobic interactions with other amino acid residues, Ile10, Asp86, Lys88, and Lys89, as shown in Figure 3B.

**Figure 4:**
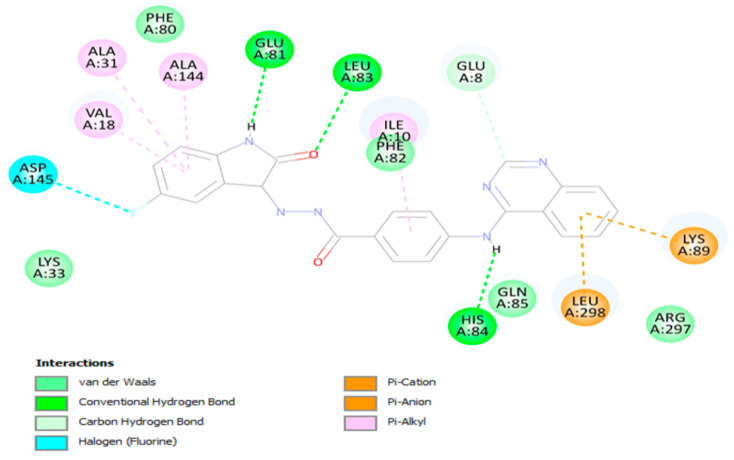
2D interaction of compound **6c** with the active site of CDK2.

Assuming that compound **6c** exerts its anticancer activity by inhibiting CDK2, by docking compound **6c** into the CDK2 active site, possible interactions and alignment between compound **6c** and the original inhibitor sunitinib were examined (Figure 4). Compound **6c** and sunitinib docked into the CDK2 active site with binding energies of −10.2 and −9 kcal/mol, respectively, indicating **6c** to have a higher possible binding affinity than that of the original ligand. The docking simulation predicted that compound **6c** is well accommodated inside the active site of CDK2 enzyme, with different interactions with Lys89, His 84, Glu8, Ile10, Leu134, and Leu83. It can also be seen that the proton-donating NH group forms a conventional H-bond with His84. The benzene part of quinazoline ring was observed to form a cation-π interaction with Lys89.

#### 2.3.2. In Silico ADME Study

The findings attained from the in silico investigation unequivocally show that compounds **6a**–**d** with different group substitutions are druggable substances showing good ADME properties. The results obtained from the SwissADME search engine are listed in Table 6.

According to the pharmacokinetic properties, compounds **6a**–**d** showed high gastrointestinal absorption and no BBB permeability; however, drug likenesses indicate that these compounds may have minimal CNS side effects. Additionally, the results suggest that all compounds may be inhibitors of the CYP3A4 enzyme while only **6a** is an inhibitor of the CYP2D6 enzyme. Finally, low Log Kp values suggest that all these compounds are likely to penetrate skin.

#### 2.3.3. Toxicity Prediction

Using the OSIRIS property explorer (https://www.organic-chemistry.org/prog/peo/), theoretical toxicity profiles (mutagenic, tumorigenic, irritant, and reproductive effective) of compounds **6a**–**d** were analyzed and compared to doxorubicin as a reference drug. According to the results shown in Table 7, all tested compounds showed no toxicity risk.

## 3. Materials and Methods

### 3.1. Instrumentation

Melting points (°C) in one-open-ended glass capillaries were recorded using an electrothermal 9200 melting point apparatus (Cole-Parmer GmbH, Wertheim, Germany) and Stuart SMP 10 digital melting point apparatus (Cole-Parmer Ltd., Altrincham, UK). Nuclear magnetic resonance (NMR) spectra were recorded (700 MHz for proton ^1^H and 176 MHz for carbon ^13^C) in deuterated dimethyl sulfoxide (DMSO-*d*_6_) as a solvent, using Bruker spectrometers (Bruker, Zurich, Switzerland) at the College of Pharmacy, King Saud University, Saudi Arabia. Tetramethylsilane (TMS) was used as an internal standard. All chemical shifts are denoted per the δ scale (ppm); coupling constants (*J*) for ^1^H are given in Hz and expressed as (s) for singlet, (brs) for broad singlet, (d) for doublet, (t) for triplet, (dd) for doublet of doublets, (td) for a triplet of doublets, and (m) for multiplet. Mass spectra were analyzed by Agilent Single Quad Mass Spectrometer (Agilent Technologies, Palo Alto, CA, USA). Elemental analyses were performed on Perkin Elmer 2400 CHN elemental analyzer (Perkin Elmer, Springfield, IL, USA). Thin-layer chromatography (TLC) was performed for monitoring the reaction and checking the purity of the final product using silica gel pre-coated aluminum sheets (60 F254, Merck, Darmstadt, Germany) and visualization with an ultraviolet (UV) lamp at 365 and 254 nm.

### 3.2. Chemicals

All materials and solvents were reagents of laboratory grade and were used without any further purification. 4-Chloroquinazoline, ethyl 4-aminobenzoate, isatin, 5-methylisatin, 5-fluoroisatin, and 5-chloroisatin were purchased from AK Scientific (Union City, CA, USA). Hydrazine hydrate 80% was purchased from Alpha Chemika (Mumbai, India). Absolute ethanol and methanol used were of laboratory grade and were purchased from Fisher Scientific (Waltham, MA, USA).

### 3.3. Chemical Synthesis

#### 3.3.1. Chemistry

##### Synthesis of Ethyl 4-[(Quinazolin-4-yl)amino]benzoate (**3**)

Ethyl 4-[(quinazolin-4-yl)amino]benzoate (**3**) was prepared by the reaction of 4-chloro quinazoline (**1**) (2.14 g, 13 mmol) with ethyl 4-aminobenzoate (**2**) (4.29 g, 26 mmol, 2 equiv) in refluxing absolute ethanol (30 mL) for 6 h as described by Kapustyansky et al. [39]. The reaction mixture was then cooled, filtered, and washed with cold ethanol to obtain **3** (2.47 g, 8.45 mmol; 68%).


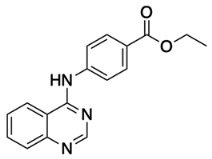


Light yellow solid, yield 68%; m.p.: 221 °C [39].

##### Synthesis of 4-[(Quinazolin-4-yl)amino] Benzohydrazide (**4**)

Reacting ethyl 4-[(quinazolin-4-yl)amino]benzoate (**3**) (2 g, 6.85 mmol) with excess hydrazine hydrate 80% in refluxing absolute ethanol for 8 h resulted in the formation of 4-[(quinazolin-4-yl)amino]benzohydrazide (**4**) (0.97g, 3.5 mmol; 51%) as white powder. The reaction was performed as described by Kovalenko, S. et al. [40].


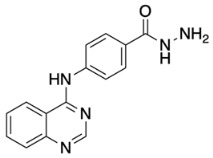


Light yellow fine powder, yield 51%; m.p.: 269 °C [40].

##### General Procedure for Synthesis of *N*′-(5-Subsitituted-2-oxoindolin-3-ylidene)-4-(quinazolin-4-ylamino) Benzohydrazide Derivatives (**6a**–**d**)

Compounds **6a**–**d** were prepared by refluxing of **4** (0.15 g, 0.5 mmol) with the corresponding isatin (1.2 equiv) in absolute ethanol treated with 0.2 mL of glacial acetic acid for 8 h. The reaction was then stopped and left to cool before it was filtered and washed with cold ethanol to obtain compounds **6a**–**d** (22–86%).

(*Z*)-*N*′-(2-Oxoindolin-3-ylidene)-4-(quinazolin-4-ylamino) benzohydrazide (**6a**).

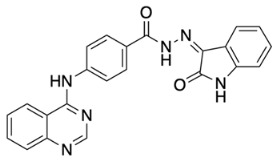


Light yellow solid, yield 30%; m.p.: 337 °C. ^1^H NMR (700 MHz, DMSO-*d*_6_) δ: 6.99 (d, 1H, *J* = 7.7 Hz), 7.14 (t, 1H, *J* = 7.7 Hz), 7.41 (t, 1H, *J* = 7.7 Hz), 7.64 (d, 1H, *J* = 7.7 Hz), 7.72 (t, 1H, *J* = 7.7 Hz), 7.87 (d, 1H, *J* = 8.4 Hz), 7.93 (t, 1H, *J* = 7.7 Hz), 7.97 (d, 2 H, *J* = 8.4 Hz), 8.22 (d, 2H, *J* = 9.1 Hz), 8.64 (d, 1H, *J* = 8.4 Hz), 8.74 (s, 1H), 10.13 (s, 1H, NH), 11.40 (s, 1H, NH), 13.99 (s, 1H, NH) ppm. ^13^C NMR (176 MHz, DMSO-*d*_6_) δ: 111.6, 115.8, 120.4, 121.4, 122.0, 123.2, 123.6, 126.6, 128.4, 132.2, 133.9, 142.8, 144.2, 150.3, 154.7, 158.0, 163.6 ppm. MS (ESI): *m*/*z* (%): 406.9 [M-1]. Anal. Calcd for C_23_H_16_N_6_O_2_: C, 67.64; H, 3.95; N, 20.58. Found: C, 67.41; H, 4.02; N, 20.64.

(Z)-*N*′-(5-Chloro-2-oxoindolin-3-ylidene)-4-(quinazolin-4-ylamino)benzohydrazide (**6b**).

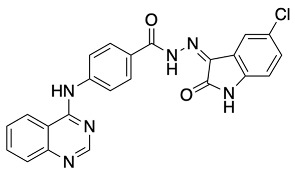


Yellow powder, yield 68%; m.p.: 361 °C. ^1^H NMR (700 MHz, DMSO-*d*_6_): δ 7.00 (d, 1H, *J* = 8.4 Hz), 7.45 (dd, 1H, *J* = 2.1, 8.4 Hz), 7.61 (d, 1H, *J* = 2.1 Hz), 7.71 (t, 1H, *J* = 7.7 Hz), 7.86 (d, 1H, *J* = 7.7 Hz), 7.93 (t, 1H, *J* = 8.4 Hz), 7.96 (d, 2H, *J* = 8.4 Hz), 8.22 (d, 2H, *J* = 8.4 Hz), 8.63 (d, 1H, *J* = 8.4 Hz), 8.74 (s, 1H), 10.13 (s, 1H, NH), 11.51 (s, 1H, NH), 13.92 (s, 1H, NH) ppm. ^13^C NMR (176 MHz, DMSO-*d*_6_): δ 113.2, 115.8, 120.9, 121.9, 122.1, 123.5, 126.4, 127.1, 127.4, 128.4, 131.5, 133.8, 141.5, 144.3, 150.3, 154.6, 157.9, 163.5 ppm. MS (ESI): *m*/*z* (%): 440.9 [M-1]. Anal. Calcd for C_23_H_15_ClN_6_O_2_: C, 62.38; H, 3.41; N, 18.98. Found: C, 62.49; H, 3.52; N, 18.77.

(*Z*)-*N*′-(5-Fluoro-2-oxoindolin-3-ylidene)-4-(quinazolin-4-ylamino)benzohydrazide (**6c**).

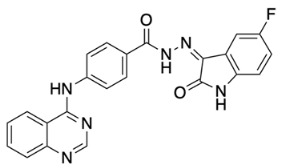


Yellow powder, yield 22%; m.p.: 333 °C. ^1^H NMR (700 MHz, DMSO-*d*_6_) δ: 6.98 (dd, 1H, *J* = 4.2, 8.4 Hz), 7.27–7.24 (tt, 1H, *J* = 2.1, 9.1 Hz), 7.46 (dd, 1H, *J* = 2.1, 8.4 Hz), 7.71 (t, 1H, *J* = 8.4 Hz), 7.86 (d, 1H, *J* = 8.4 Hz), 7.93 (m, 1H), 7.96 (d, 2H, *J* = 8.4 Hz), 8.22 (d, 2H, *J* = 9.1 Hz), 8.64 (d, 1H, *J* = 8.4 Hz), 8.74 (s, 1H), 10.13 (s, 1H, NH), 11.42 (s, 1H, NH), 13.99 (s, 1H, NH) ppm. ^13^C NMR (176 MHz, DMSO-*d*_6_): δ 108.5 (d, JC-F = 26.4 Hz), 112.8 (d, JC-F = 8.8 Hz), 115.8, 118.5 (d, JC-F = 24.6 Hz), 121.7 (d, JC-F = 8.8 Hz), 121.9, 123.6, 126.4, 127.1, 128.4, 133.8, 139.1, 144.3, 150.3, 154.7, 157.9, 158.9 (d, JC-F = 244.6 Hz), 163.7 ppm. MS (ESI): *m*/*z* (%): 425 [M-1]. Anal. Calcd for C_23_H_15_FN_6_O_2_: C, 64.79; H, 3.55; N, 19.71. Found: C, 65.02; H, 3.48; N, 19.82.

(*Z*)-*N*′-(5-Methyl-2-oxoindolin-3-ylidene)-4-(quinazolin-4-ylamino)benzohydrazide (**6d**).

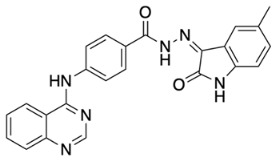


Yellow powder, yield 87%; m.p.: 348 °C. ^1^H NMR (700 MHz, DMSO-*d*_6_) δ: 2.33 (s, 3H, CH_3_), 6.88 (d, 1H, *J =* 7.7 Hz), 7.22 (d, 1H, *J =* 7.7 Hz), 7.46 (s, 1H), 7.72 (m, 1H), 7.86 (d, 1H, *J =* 8.4 Hz), 7.93 (m, 1H), 7.96 (d, 2H, *J =* 8.4 Hz), 8.22 (d, 2H, *J =* 8.4 Hz), 8.65 (d, 1H, *J =* 7.7 Hz), 8.74 (s, 1H), 10.21 (s, 1H, NH), 11.29 (s, 1H, NH), 13.99 (s, 1H, NH) ppm. ^13^C NMR (176 MHz, DMSO-*d*_6_): δ 21.0 (CH_3_), 111.5, 115.8, 120.4, 121.7, 121.9, 123.6, 126.7, 127.1, 128.4, 132.3, 132.6, 133.9, 140.6, 144.2, 150.3, 154.7, 158.0, 163.8 ppm. MS (ESI): *m*/*z* (%): 420.9 [M-1]. Anal. Calcd for C_24_H_18_N_6_O_2_: C, 68.24; H, 4.29; N, 19.89. Found: C, 68.15; H, 4.38; N, 19.96.

### 3.4. Biological Screening

#### 3.4.1. Cell Culture

The four cancer cell lines—hepatocellular carcinoma (HepG2), breast adenocarcinoma (MCF-7), breast adenocarcinoma (MDA-MB-231), and cervical carcinoma (HeLa)—and the normal fibroblast cell line (WI38) used in this study were obtained from the American Type Culture Collection (ATCC, Manassas, VA, USA) via a holding company for biological products and vaccines (VACSERA, Cairo, Egypt). Antibiotic solutions and sterile solutions were purchased from Sigma-Aldrich (St. Louis, MO, USA).

#### 3.4.2. Cytotoxicity Assay

Cytotoxicity of the synthesized compounds **6a**–**d** was assessed by performing a colorimetric MTT (3-(4,5-dimethylthiazol-2-yl)-2,5-diphenyltetrazolium bromide) assay [41,42,43]. The MTT assay is widely used to measure cell proliferation, cell viability, and cytotoxicity. It is based on reducing MTT dye into formazan crystals by living cells, determining mitochondrial activity [44]. Experimental procedures are described in detail in the Supplementary Information.

#### 3.4.3. Flow Cytometry Cell Cycle Analysis

Cell cycle arrest and distribution were evaluated using a Propidium Iodide Flow Cytometry Kit (ab139418, Abcam, Cambridge, MA, USA) followed by flow cytometry analysis [45,46]. Experimental procedures are described in detail in the Supplementary Information.

#### 3.4.4. Annexin V-FITC Dual-Staining Apoptosis Assay

The apoptosis assay was performed with an annexin V-FITC/PI double staining apoptosis detection kit (K101, BioVision Inc., Mountain View, CA, USA) using a flow cytometer [47,48]. Experimental procedures are described in detail in theSupplementary Information.

#### 3.4.5. Gene Expression Analysis

RNA was isolated from cells using RNeasy^®^ (Qiagen, Chatsworth, CA, USA) [49]. Experimental procedures are described in detail in the Supplementary Information.

#### 3.4.6. In Vitro CDK2, EGFR, VEGFR-2, and HER2 Inhibitory Assays

The in vitro inhibitory activities of the compound **6c** against CDK2, EGFR, VEGFR-2, and HER2 were carried out using a CDK2, EGFR, VEGFR-2, and HER2 Kinase Assay Kit (BPS Biosciences, San Diego, CA, USA) [50]. Experimental procedures are described in detail in the Supplementary Information.

### 3.5. In Silico Study

#### 3.5.1. Molecular Docking

The protein crystal structure data was acquired from the Protein Data bank (PDB). The 3D crystal structure used for docking was 3TI1 (CDK2 in complex with Sunitinib). The docking study was conducted using Autodock Vina in PyRx (version 0.8) virtual screening tool (http://pyrx.sourceforge.net), AutoDock Tools (version 1.5.7), and BIOVIA Discovery Studio Visualizer 2021 (version 24.1.0.23298) [51]. Procedures are described in detail in the Supplementary Information.

#### 3.5.2. In Silico ADME Study

The ADME study was carried out using the SwissADME predictor (http://www.swissadme.ch/, accessed on 21 May 2021), which is a free web tool to estimate pharmacokinetics, and drug likeness. The SMILES notations used were generated from the synthesized compounds.

#### 3.5.3. Toxicity Prediction

Osiris Property Explorer (http://www.organic-chemistry.org/prog/peo/, accessed on 25 May 2021) was used to determine toxicity risk. The results of virtual screening were evaluated and color-coded for properties such as potential mutagenicity, reproductive system, irritant effect, and tumorigenicity. This program’s predictions are based on functional group similarities of the investigated compounds with those in its database that have been extensively studied in vitro and in vivo.

## 4. Conclusions

In summary, four isatin–quinazoline compounds **6a**–**d** were synthesized through three steps. The target compounds were obtained with different yields. Most of the synthesized compounds were found to suppress proliferation and induce cytotoxicity toward HepG2, MCF-7, MDA-MB-231, and HeLa cell lines, in varying degrees compared with standard drugs doxorubicin and sunitinib. In particular, compounds **6a** and **6c** showed significant cytotoxic activity, with **6c** being the most active. Compound **6c** was shown to induce cell cycle arrest in the sub-G1 and S phases of the HepG2 cell line. Moreover, in vitro inhibitory activity of compound **6c** on CDK2, EGFR, VEGFR-2, and HER2 protein kinase enzymes was evaluated. Compound **6c** demonstrated significant inhibitory activity toward those protein kinase enzymes compared to the reference drugs. Additionally, the annexin V-FITC/PI assay showed that compounds **6c** had an early apoptotic induction effect in cell cycle assays. Furthermore, this compound caused a notable downregulation of the Bcl-2 protein level, a notable elevation of the Bax protein level, and a considerable rise in the activities of caspase-3 and caspase-9. Lastly, a molecular docking investigation of **6c**’s interactions with the CDK2 active pocket via several interactions supporting the results obtained. Overall, these findings suggest that compound **6c** is a promising multi-kinase inhibitor and candidate anticancer drug.

## Figures and Tables

**Table 1:** Growth inhibitory activity (IC_50_ μM) of the tested compounds against HepG2, MCF-7, MDA-MB-231, and HeLa cancer cells.

Compound	IC_50_ (µM)				
	WI38	HepG2	MCF-7	MDA-MB-231	HeLa
Sunitinib	43 ± 2.9	6.82 ± 0.5	5.19 ± 0.4	8.41 ± 0.7	7.48 ± 0.6
Doxorubicin	6.72 ± 0.5	4.50 ± 0.2	4.17 ± 0.2	3.18 ± 0.1	5.57 ± 0.4
**6a**	34.07 ± 2.6	7.98 ± 0.6	4.89 ± 0.3	6.24 ± 0.5	13.07 ± 0.9
**6b**	>100	61.35 ± 3.5	70.65 ± 3.4	56.31 ± 3.2	66.82 ± 3.4
**6c**	57.28 ± 3.3	2.61 ± 0.1	3.78 ± 0.2	5.18 ± 0.4	7.60 ± 0.5
**6d**	65.83 ± 3.8	87.41 ± 4.5	>100	82.67 ± 4.3	>100

**Table 2:** Cell cycle analysis after 24 h incubation with compound **6c**.

Sample	Cell Cycle Distribution (%)			
	%G0–G1	%S	%G2/M	% Sub-G1
HepG2	46.85	39.28	13.87	1.73
**6c/**HepG2	36.47	53.61	9.92	42.79

**Table 3:** Apoptosis induction analysis.

Sample	Apoptosis Induction Analysis			Necrosis
	Total	Early	Late	
HepG2 Control	1.73	0.42	0.22	1.09
**6c/**HepG2	42.79	28.64	11.21	2.94

**Table 4:** Effect of compound **6c** on levels of Bax, Bcl-2, caspase-9, and caspase-3.

Sample	Protein Expression (Normalized to β-Actin)			
	Bax	Bcl-2	Caspase-3	Caspase-9
HepG2	1	1	1	1
Staurosporine/HepG2	6.794	0.351	8.43	4.614
**6c/**HepG2	4.585	0.185	10.13	2.795

**Table 5:** Inhibitory activities of **6c** against CDK2, EGFR, VEGFR-2, and HER2 protein kinase.

Protein Kinase	Compound	IC_50_ (μM)
CDK2	**6c**	0.183 ± 0.01
	Roscovitine	0.595 ± 0.031
EGFR	**6c**	0.083± 0.005
	Lapatinib	0.043 ± 0.003
VEGFR-2	**6c**	0.076 ± 0.004
	Sorafenib	0.045 ± 0.002
HER2	**6c**	0.138 ± 0.07
	Lapatinib	0.06 ± 0.003

**Table 6:** Predictions of possible pharmacokinetics and drug-likeness properties for compounds **6a**–**d** using SwissADME.

Compound	Pharmacokinetics						Drug Likeness (#Violations)					
	GI Absorption	BBB Permeant	Pgp Substrate	CYP2D6 Inhibitor	CYP3A4 Inhibitor	log Kp (Skin Permeation) cm/s	Lipinski	Ghose	Veber	Egan	Muegge	Bioavailability Score
**6a**	High	No	No	Yes	Yes	−5.8	0	0	0	0	0	0.55
**6b**	High	No	No	No	Yes	−5.57	0	0	0	0	0	0.55
**6c**	High	No	No	No	Yes	−5.84	0	0	0	0	0	0.55
**6d**	High	No	No	No	Yes	−5.63	0	0	0	0	0	0.55

**Table 7:** Predicted toxicity effects using OSIRIS property explorer.

Compound	Toxicity Risk			
	Mutagenic	Tumorigenic	Irritant Effect	Reproductive Effective
**6a**	None	None	None	None
**6b**	None	None	None	None
**6c**	None	None	None	None
**6d**	None	None	None	None
Doxorubicin	None	None	None	High

## Data Availability

Data is contained within the article or Supplementary Material.

## Supplementary Materials

The following supporting information can be downloaded at: https://www.mdpi.com/article/10.3390/ph18101546/s1, ^1^H NMR, ^13^C NMR and Mass spectra.
