# Supplementary material for: Multi-Kinase Inhibition by New Quinazoline–Isatin Hybrids: Design, Synthesis, Biological Evaluation and Mechanistic Studies
Source: Pharmaceuticals (Basel). 2025 Oct 14;18(10):1546. doi: 10.3390/ph18101546 (PMC12567098; doi:10.3390/ph18101546)
Supplement: Supplementary file 1 [file pharmaceuticals-18-01546-s001.zip › pharmaceuticals-3917227-supplementary.pdf]

Supporting Information

Multi-kinase Inhibition of New Quinazoline-isatin Hybrids: Design, Synthesis, Biological Evaluation and Mechanistic Studies

Mohammed M. Alanazi* and Reem I. Al-wabli*

Department of Pharmaceutical Chemistry, College of Pharmacy, King Saud University, Riyadh 11451, Saudi Arabia

Correspondence: mmalanazi@ksu.edu.sa and ralwabli@ksu.edu.sa

3.4. Biological Screening

3.4.1. Cell culture

Four cancer cell lines; Hepatocellular carcinoma (HepG2), Breast adenocarcinoma (MCF-7), Breast adenocarcinoma (MDA-MB-231), and Cervical carcinoma (HeLa) and normal fibroblast cell line (WI38) used in this study were obtained from American Type Culture Collection (ATCC, USA) via Holding company for biological products and vaccines (VACSERA), Cairo, Egypt. Antibiotic solutions and sterile solutions were purchased from Sigma-Aldrich.

3.4.2. Cytotoxicity Assay

Cytotoxicity of the synthesized compounds **6a-d** was done by performing a colorimetric MTT (3-(4,5-dimethylthiazol-2-yl)-2,5-diphenyltetrazolium bromide) assay.[41–43] MTT assay is widely used to measure cell proliferation, cell viability, and cytotoxicity. It is based on reducing MTT dye into formazan crystals by living cells, determining mitochondrial activity.[44] For the assay, cells were seeded in Dulbecco's modified minimum essential medium (DMEM) at a density of 1.0×10^4 cells/well in 96-well plates, supplemented with 10% FBS and 5% CO_2 at 37 °C for 24 h. To each well, a test compound was added at different concentrations. Each concentration was examined three times. Then the cells were treated with the compounds at 37 °C under 5 % CO_2 for 48 h. Following this, MTT dye was added to each well at a final concentration of 0.5 mg/ml. Cells were treated with this tetrazolium dye for 4 h. Next, purple crystals of formazan were noticed in each well, formed as a metabolic product of MTT. These crystals were dissolved by adding 100 µl dimethyl sulfoxide (DMSO), and the absorbance in each well was recorded at 570 nm in a microplate reader (EXL 800, USA). Absorbance at 570nm directly correlates with cell viability. IC_{50} (µM) values were determined using Graph Pad Prism software version 5 (Graph Pad Software Inc, CA). The cells were also treated with Doxorubicin; a standard anticancer drug used as positive control.

3.4.3. Flow cytometry cell cycle analysis

Cell cycle arrest and distribution were evaluated using the Propidium Iodide Flow Cytometry Kit (ab139418, Abcam) followed by flow cytometry analysis.[45,46] First, HepG2 cells were cultured at a density of $2 \times 10^{5/}$well for 24 h in the presence of tested newly synthesized compounds **6c** (2.6 µM) (control cells were left untreated and both measurements were done in triplicate). Then, the attached cells were supplemented with trypsin, washed with ice-cold PBS, centrifuged, and fixed using ice-cold 70% ethanol. Next, cells were re-suspended and stored at $^{+}4$ ^{o}C for at least 2 h. After that, cells were incubated with 200 µL 1X Propidium Iodide (PI) + RNase Staining Solution for 30 min at 37 ^{o}C in the dark. The DNA content in each cell nucleus was determined by Epics XL-MCL™ flow cytometer (Beckman- Coulter, USA). Finally, cell cycle phase distribution was analyzed using Flowing software 2.5.1 (Turku Centre for Biotechnology, University of Turku, Turku, Finland).

3.4.4. Annexin V-FITC Dual-Staining Apoptosis Assay

Apoptosis assay was performed with an Annexin V-FITC / PI double staining apoptosis detection kit (K101, BioVision Inc, Mountain View, CA, USA) using a flow cytometer.[47,48] HepG2 cells were exposed to 6c at the IC50 concentration (2.6 µM) for 24 h. After treatment, the cells were collected, washed two times with PBS, and resuspended in 500 µL binding buffer. Next, annexin V-FITC (5 µL) and propidium iodide (PI) 5 µL were intruduced to the suspension and the cells were left in the dark for 15 min at room temperature. Samples were then recorded by Epics XL-MCL™ flow cytometer (Beckman- Coulter, USA) and Flowing software (Turku Centre for Biotechnology, University of Turku, Turku, Finland).

3.4.5. Gene Expression Analysis

The RNA was isolated from cells using RNeasy® (Qiagen, Chatsworth, CA, USA).[49] After the RNA extraction, one-step RT-qPCR was performed using iScript™ One-Step RT-PCR kit with SYBR® Green (Bio-Rad Inc., CA, USA) which was added to the Master Mix which contained 25 µL 2x SYBR Green®, 1.5 µL forward primer (10 µM), 1.5 µL reverse primer (10 µM), 11 µL nuclease-free water, and 1 µL ×50 iScript Reverse Transcriptase in a final reaction volume of 50 µL. Two control reactions were prepared: one without the RNA template and the other without the reverse transcriptase enzyme. The one-step RT-qPCR reaction comprised an initial incubation with the complete reaction mixture, including the iScript Reverse Transcriptase enzyme, followed by enzyme inactivation, the qPCR cycling steps (denaturation, annealing, and extension), and a final dissociation step. The amplification reactions were carried out using the Rotor-Gene Q Real-Time PCR system (Qiagen, Hilden, Germany). The expression levels were denoted as n-fold differences relative to the reference gene, β-Actin. The data were analyzed using a comparative threshold (Ct) method, and the fold inductions of samples were compared with the untreated samples. Then, the gene expression level was calculated using following formula: $2^{\Delta\Delta Ct} = 2^{Ct(\text{treated cells}) - Ct(\text{control cells})}$, where 2 is the amplification efficiency where the template doubles in each cycle during exponential amplification.

3.4.6. In vitro CDK2, EGFR, VEGFR-2, and HER2 Inhibitory Assays

The in vitro inhibitory activities of the compound 6d against CDK2, EGFR, VEGFR-2, and HER2 were carried out using CDK2, EGFR, VEGFR-2, and HER2 Kinase Assay Kit (BPS Biosciences, San Diego, CA).[50] At first, CDK2, EGFR, VEGFR-2, and HER2 and their substrates were treated with compound 6d in enzymatic buffer for 40 min at 30 oC to initi-ate the catalytic function of the enzymes. Detection reagent (Kinase-Glo® Max reagent) was the added to stop the reaction, followed by incubation at room temperature for 15 min. Then the IC50 values were calculated by measuring luminescence signal at 450 nm using a plate reader (Tecan Spark® 10M, Männedorf, Switzerland). All assays were carried out based on protocol from manufacture. All samples and controls were tested in duplicate.

3.5. In silico study

3.5.1. Molecular docking

3.5.1.1. Accession and preparation of the target protein

The protein crystal structure data was acquired from the Protein Data bank (PDB). The 3D-crystal structure used for docking is 3TI1 (CDK2 in complex with Sunitinib). The docking study was conducted using the virtual screening software (version 0.8), AutoDock Tools (version 1.5.7), and BIOVIA Discovery Studio Visualizer 2021 (version 24.1.0.23298). The protein crystal structure data was processed by eliminating all extra molecules such as water, and ligand. Then, saved as PDB file format. In AutoDock Tools the entire protein structure was protonated, and the file was saved in PDBQT format. Lastly, the co-crystallized ligand was isolated and saved in PDB format using Discovery Studio.

3.5.1.2. Ligand preparation

Compounds' chemical structures were saved in PDB file format. The PDB format was then converted to a PDBQT file using PyRx (The Scripps Research Institute, La Jolla, CA, USA) to generate atomic coordinates.

3.5.1.3. Analysis of target active binding sites

The active sites are the ligand coordinates in the original target protein grid; therefore, a grid box was employed to reduce non-specific binding interactions, minimize processing time, and define the docking size and dimension. Finally, docking simulations were performed out using PyRx 0.8, AutoDock Vina option based on scoring functions; the lowest energy poses of the examined compound were compared with the original ligand poses.

3.5.2. In silico ADME study

The ADME study was carried out using SwissADME predictor (http://www.swissadme.ch/). It is a free web tool to estimate pharmacokinetics, and drug likeness. SMILES notations were generated from the synthesized compounds and used

3.5.3. Toxicity Prediction

Osiris Property Explorer (http://www.organic- chemistry.org/prog/peo/) was used to determine toxicity risk. The results of virtual screening are evaluated and color-coded for properties such as potential mutagenicity, reproductive system, irritant effect and tumorigenicity. This program predicts based on functional group similarity of investigated compounds with the extensively in-vitro and in-vivo studied compounds present in its database.

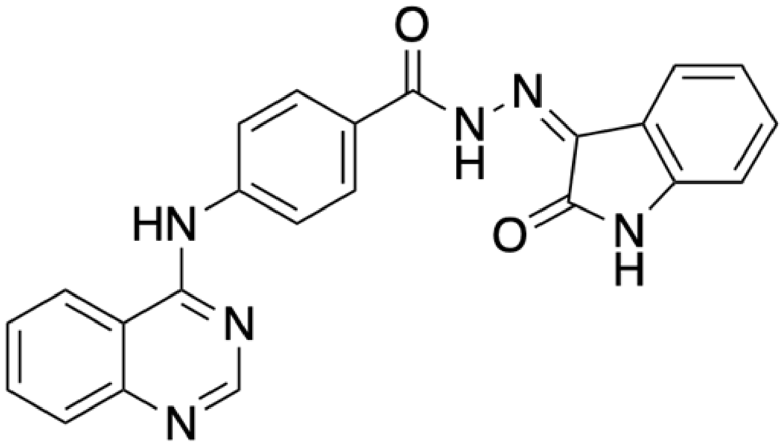
O
N
N
H
HN
O
N
H
N
N

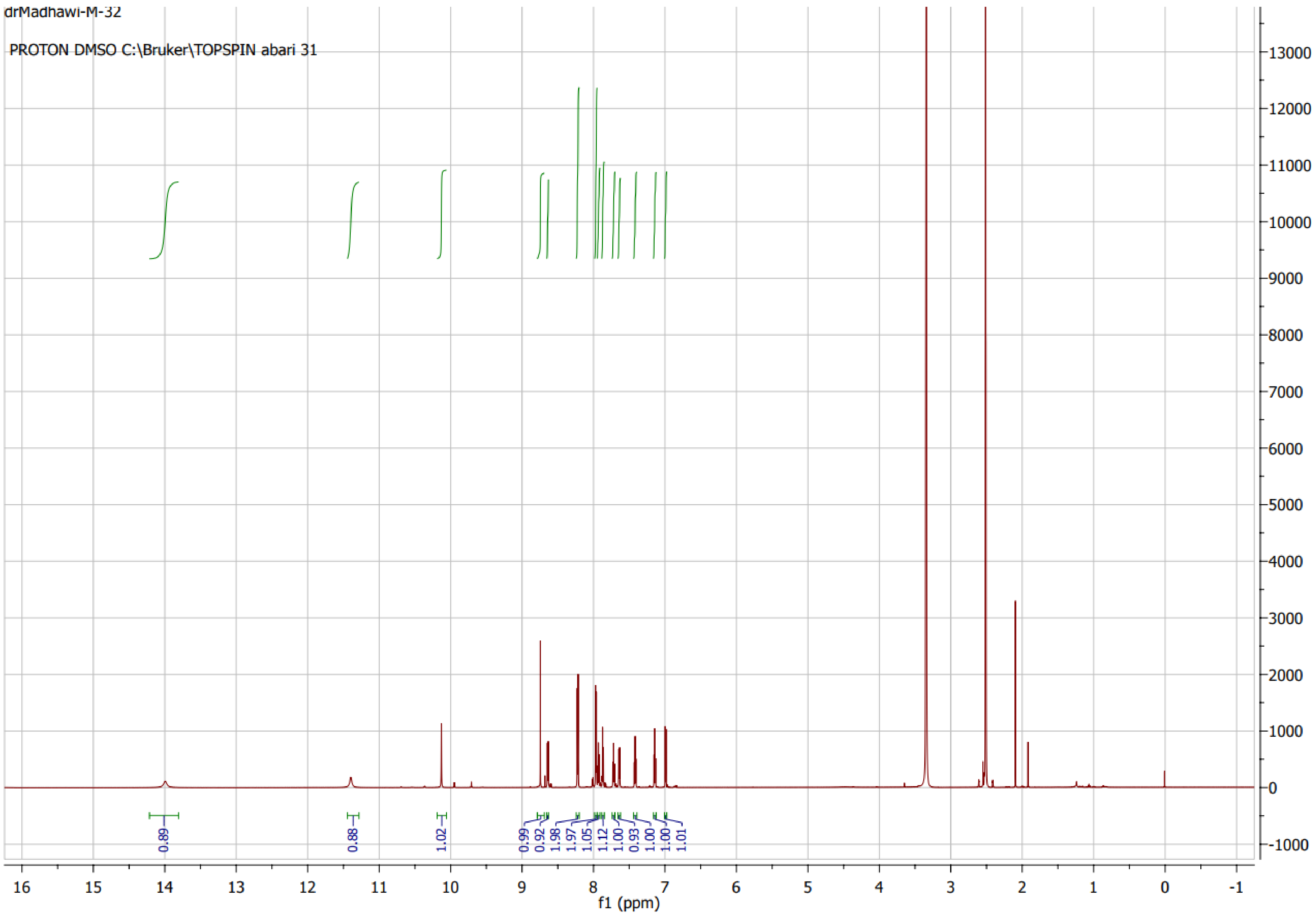

drMadhawi-M-32
PROTON DMSO C:\Bruker\TOPSPIN abari 31
0.89
0.88
1.02
0.99
0.92
1.98
1.97
1.05
1.12
1.00
0.93
1.00
1.00
1.01
16 15 14 13 12 11 10 9 8 7 6 5 4 3 2 1 0 -1
f1 (ppm)
13000 12000 11000 10000 9000 8000 7000 6000 5000 4000 3000 2000 1000 0 -1000

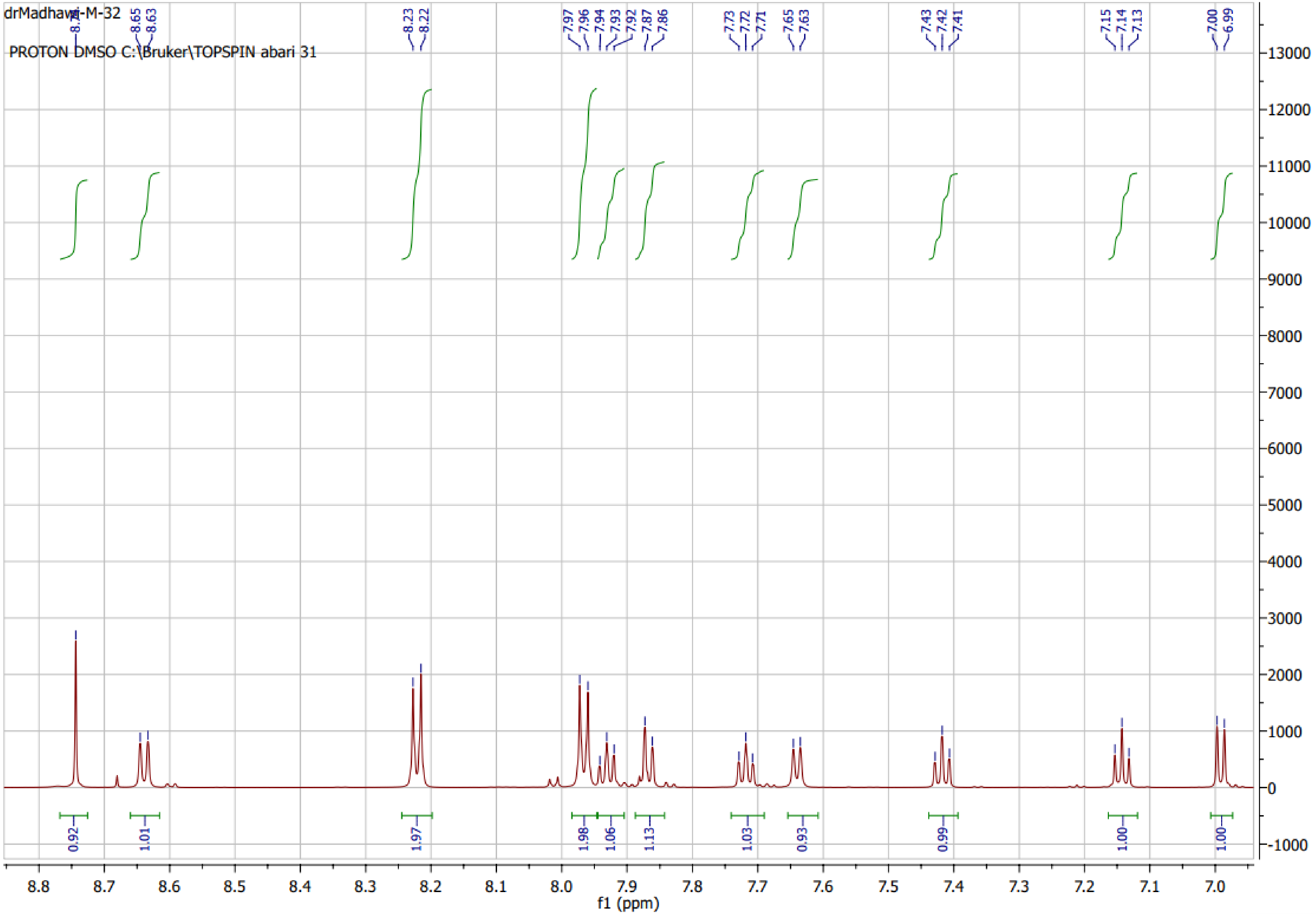
drMadhavi-M-32
PROTON DMSO C:\Bruker\TOPSPIN abari 31
8.65
8.63
8.23
8.22
7.97
7.96
7.94
7.93
7.92
7.87
7.86
7.73
7.72
7.71
7.65
7.63
7.43
7.42
7.41
7.15
7.14
7.13
7.00
6.99
0.92
1.01
1.97
1.98
1.06
1.13
1.03
0.93
0.99
1.00
1.00
f1 (ppm)

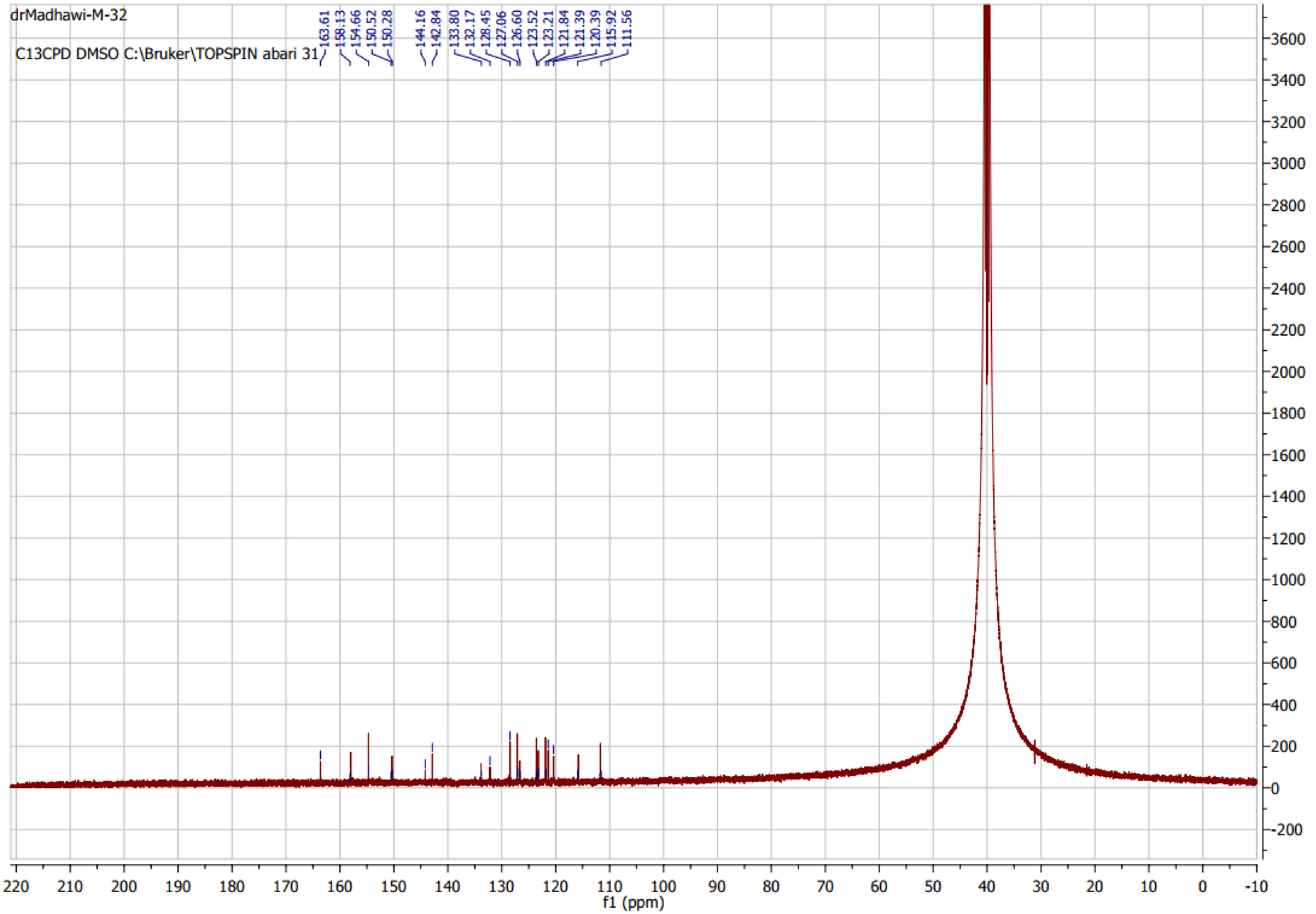

drMadhawi-M-32
C13CPD DMSO C:\Bruker\TOPSPIN abari 31
163.61
158.13
154.66
150.52
150.28
144.16
142.84
133.80
132.17
128.45
127.06
126.60
123.52
123.21
121.84
121.39
120.39
115.92
111.56
f1 (ppm)

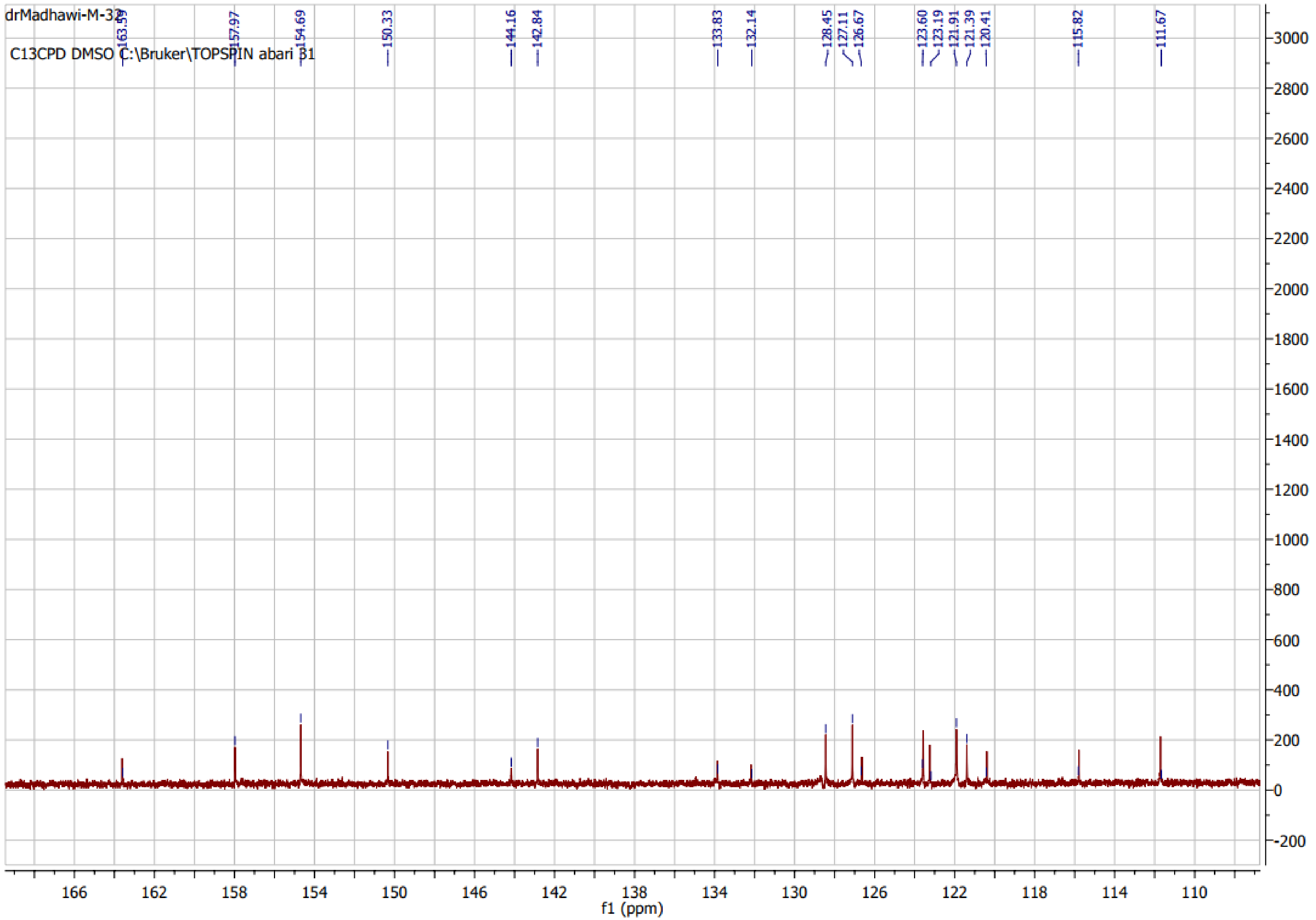
drMadhawi-M-32
C13CPD DMSO C:\Bruker\TOPSPIN abari 31
163.39
157.97
154.69
150.33
144.16
142.84
133.83
132.14
128.45
127.11
126.67
123.60
123.19
121.91
121.39
120.41
115.82
111.67
f1 (ppm)

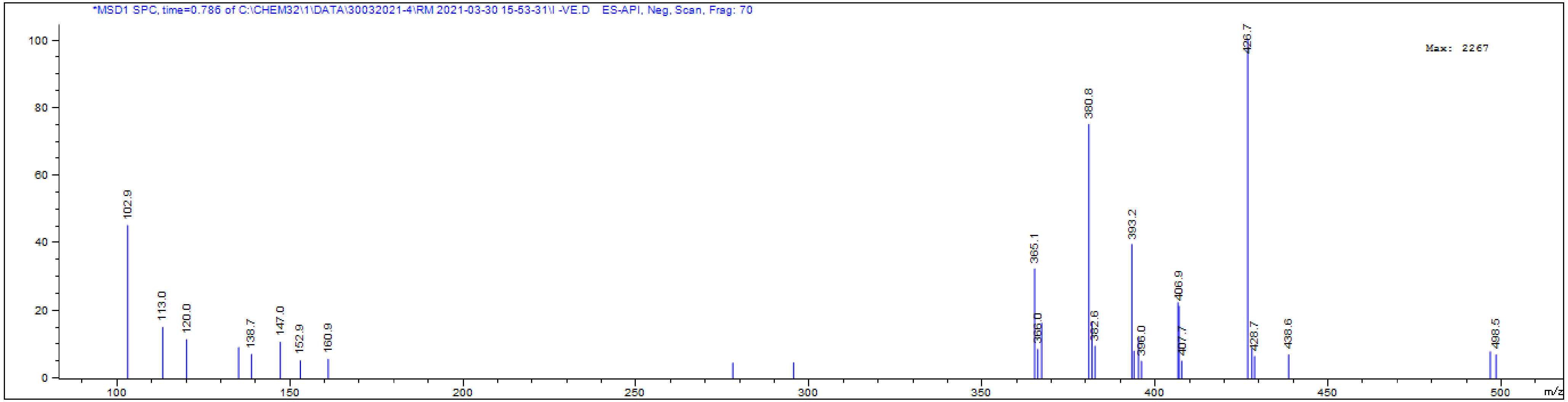

*MSD1 SPC, time=0.786 of C:\CHEM32\1\DATA\30032021-4\RM 2021-03-30 15-53-31\I -VE.D ES-API, Neg, Scan, Frag: 70
Max: 2267
102.9
113.0
120.0
138.7
147.0
152.9
160.9
365.1
366.0
380.8
382.6
393.2
396.0
406.9
407.7
426.7
428.7
438.6
498.5
m/z

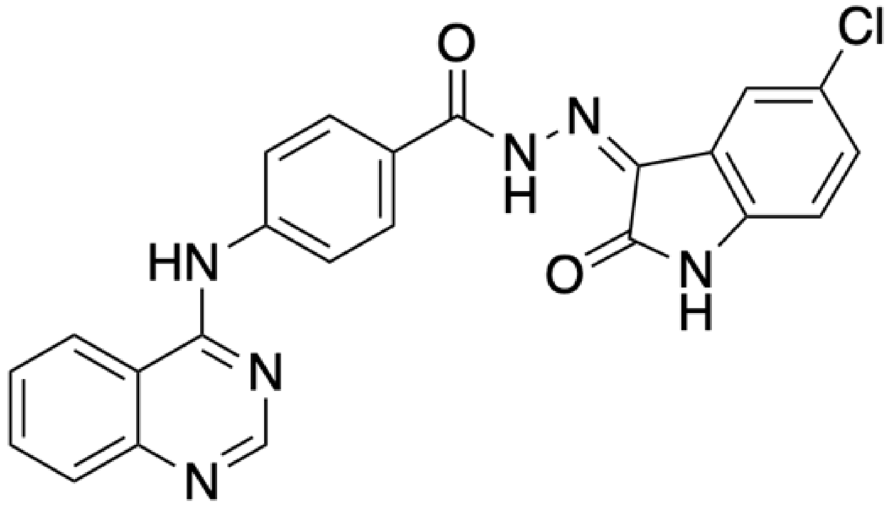

O
N
NH
Cl
HN
O
NH
N
N

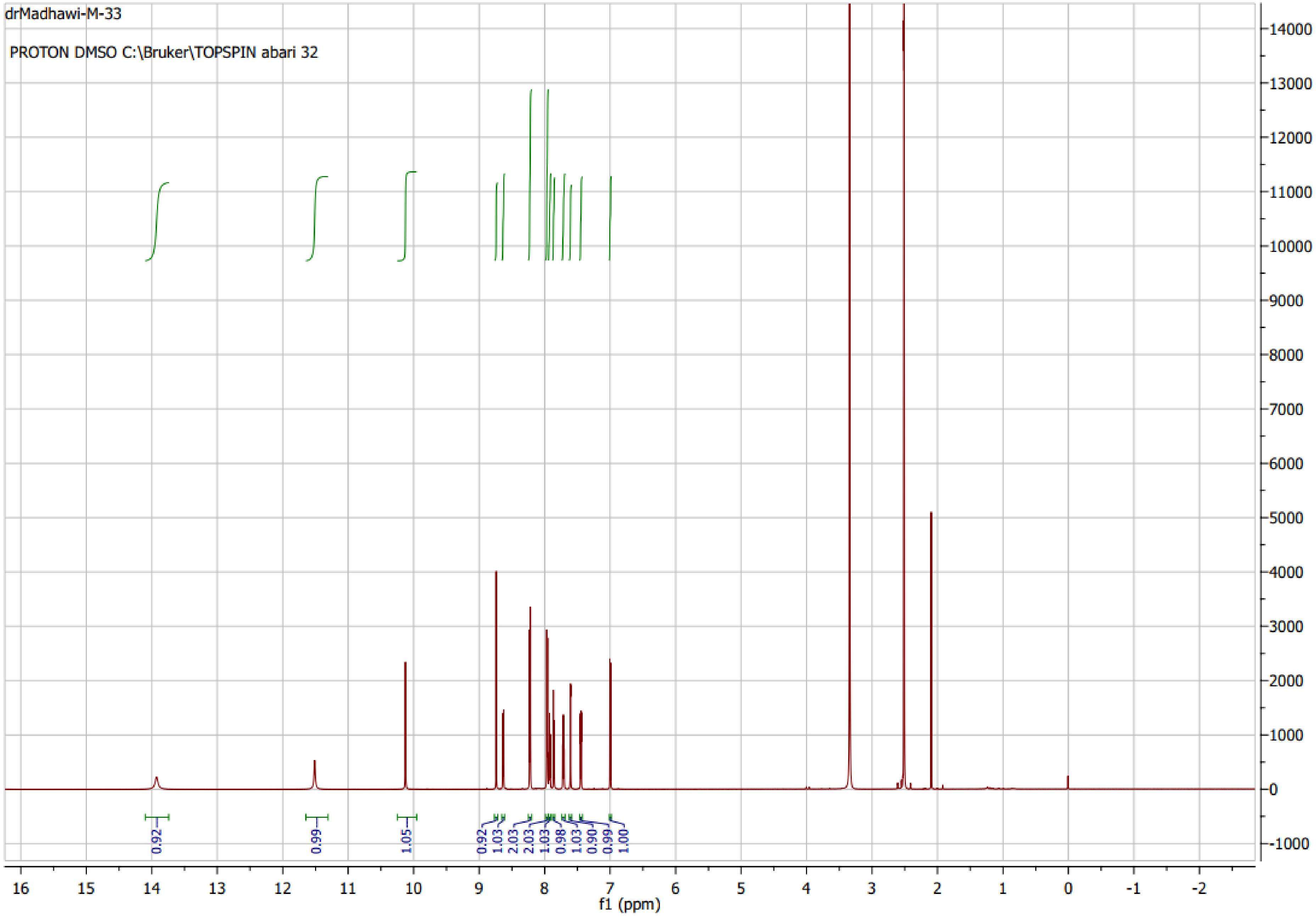
drMadhawi-M-33
PROTON DMSO C:\Bruker\TOPSPIN abari 32
14000
13000
12000
11000
10000
9000
8000
7000
6000
5000
4000
3000
2000
1000
0
-1000
0.92
0.99
1.05
0.92
1.03
2.03
2.03
1.03
0.98
1.03
0.90
0.99
1.00
16
15
14
13
12
11
10
9
8
7
6
5
4
3
2
1
0
-1
-2
f1 (ppm)

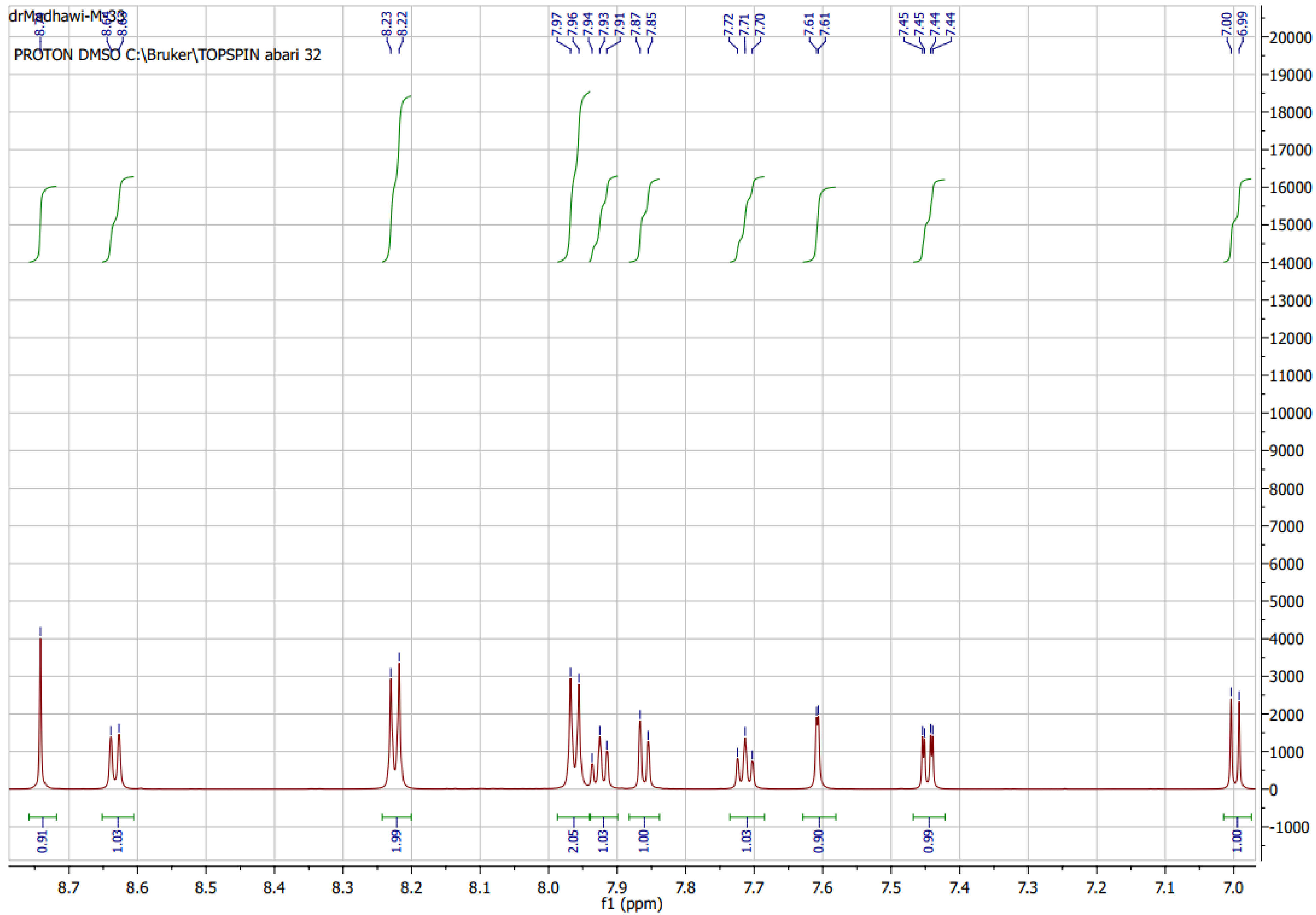
drMadhawi-M33
PROTON DMSO C:\Bruker\TOPSPIN abari 32
8.23
8.22
7.97
7.96
7.94
7.93
7.91
7.87
7.85
7.72
7.71
7.70
7.61
7.61
7.45
7.45
7.44
7.44
7.00
6.99
0.91
1.03
1.99
2.05
1.03
1.00
1.03
0.90
0.99
1.00
f1 (ppm)

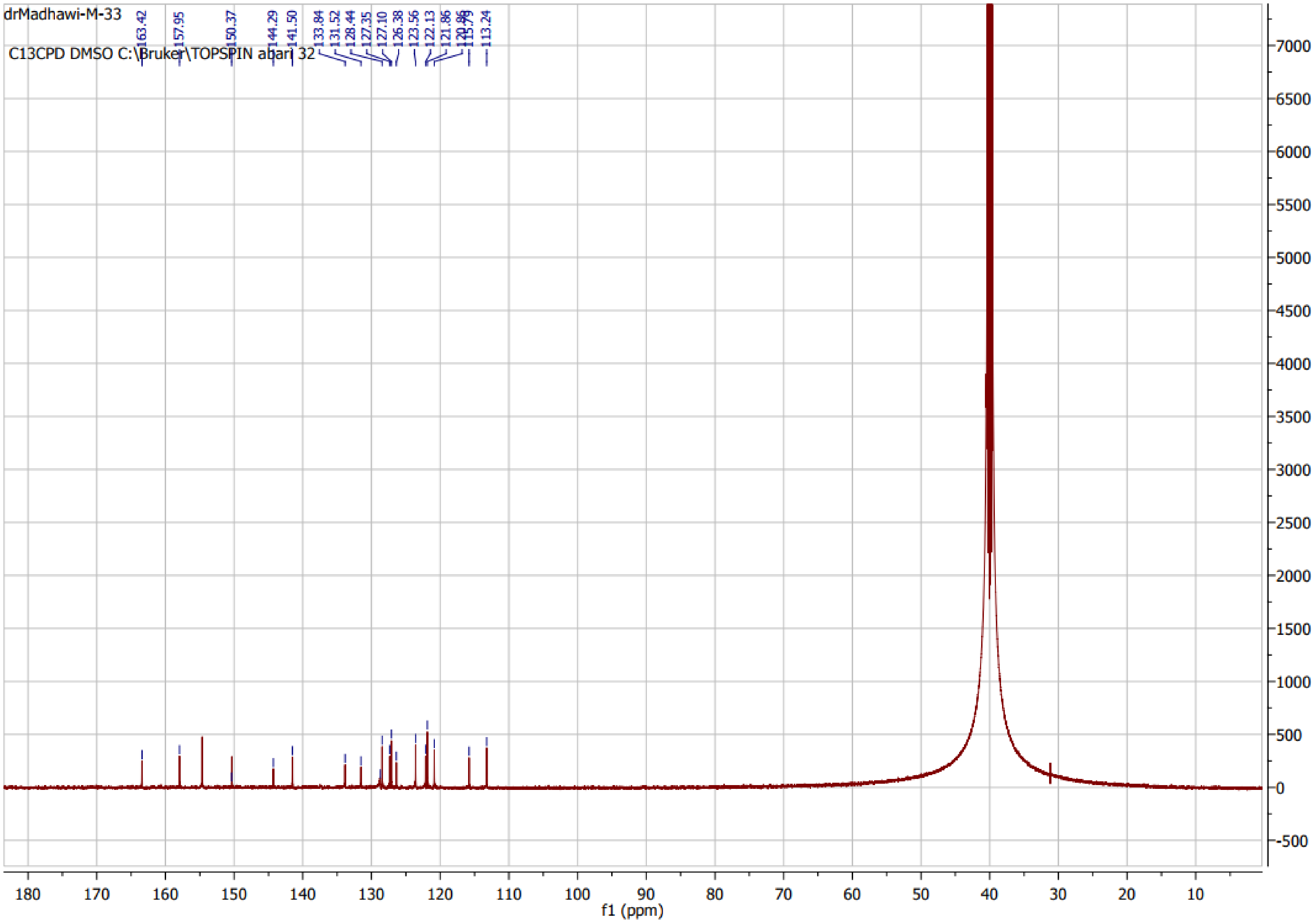
drMadhawi-M-33
C13CPD DMSO C:\Bruker\TOPSPIN abari 32
163.42
157.95
150.37
144.29
141.50
133.84
131.52
128.44
127.35
127.10
126.38
123.56
122.13
121.86
115.79
113.24
f1 (ppm)

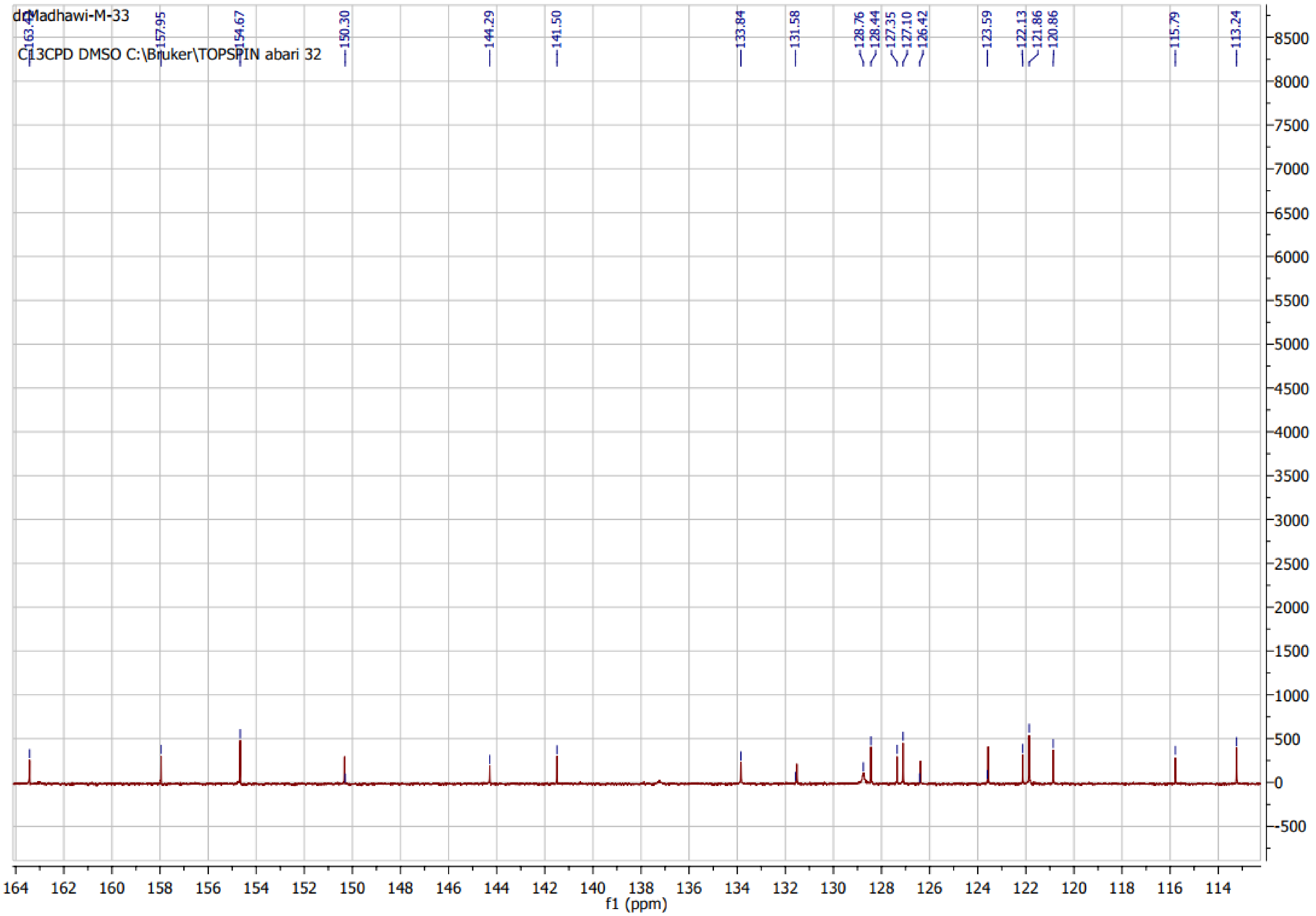
drMadhawi-M-33
C13CPD DMSO C:\Bruker\TOPSPIN abari 32
163.4[illegible]
157.95
154.67
150.30
144.29
141.50
133.84
131.58
128.76
128.44
127.35
127.10
126.42
123.59
122.13
121.86
120.86
115.79
113.24
f1 (ppm)

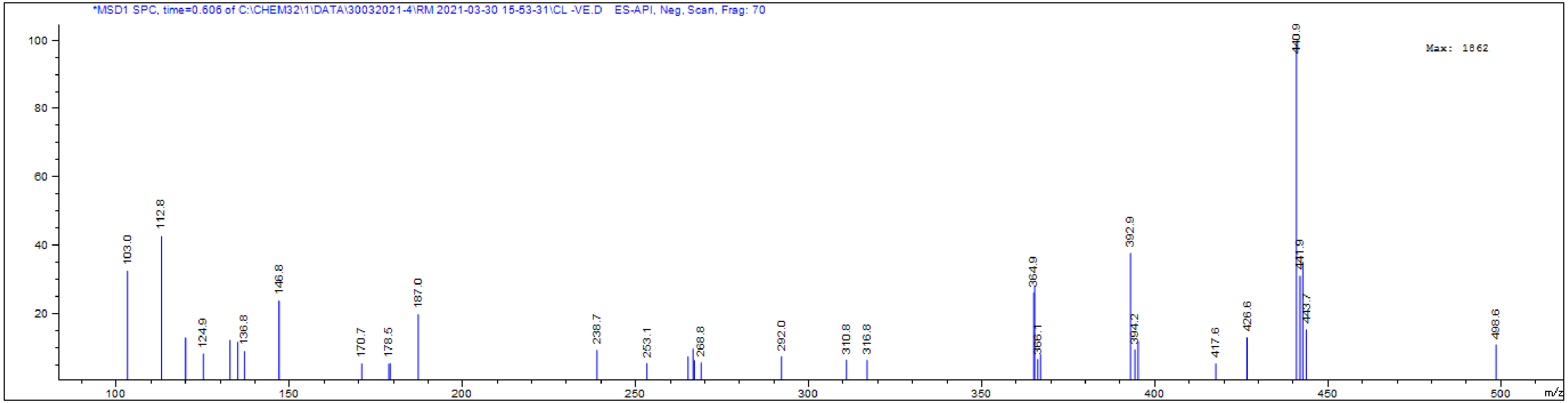

*MSD1 SPC, time=0.606 of C:\CHEM32\1\DATA\30032021-4\RM 2021-03-30 15-53-31\CL -VE.D ES-API, Neg, Scan, Frag: 70
Max: 1862
103.0
112.8
124.9
136.8
146.8
170.7
178.5
187.0
238.7
253.1
268.8
292.0
310.8
316.8
364.9
366.1
392.9
394.2
417.6
426.6
440.9
441.9
443.7
498.6
m/z

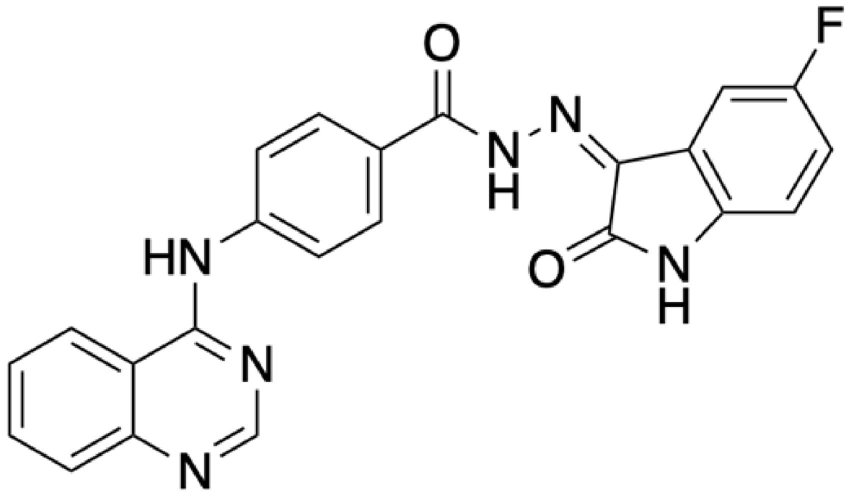
O
N
F
N
H
HN
O
N
H
N
N

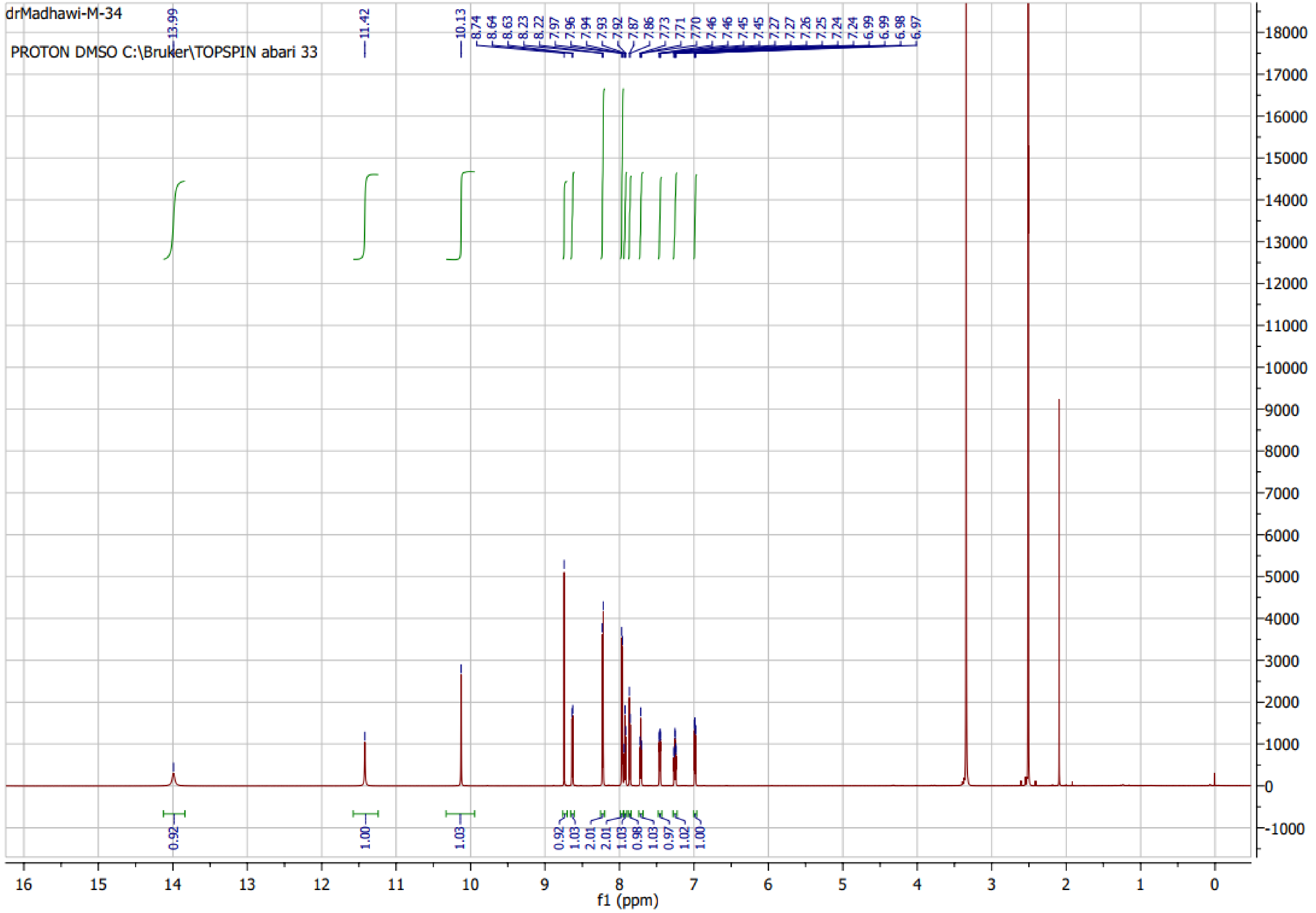

drMadhawi-M-34
PROTON DMSO C:\Bruker\TOPSPIN abari 33
13.99
11.42
10.13
8.74
8.64
8.63
8.23
8.22
7.97
7.96
7.94
7.93
7.92
7.87
7.86
7.73
7.71
7.70
7.46
7.46
7.45
7.45
7.27
7.27
7.26
7.25
7.24
7.24
6.99
6.99
6.98
6.97
0.92
1.00
1.03
0.92
1.03
2.01
2.01
1.03
0.98
1.03
0.97
1.02
1.00
f1 (ppm)

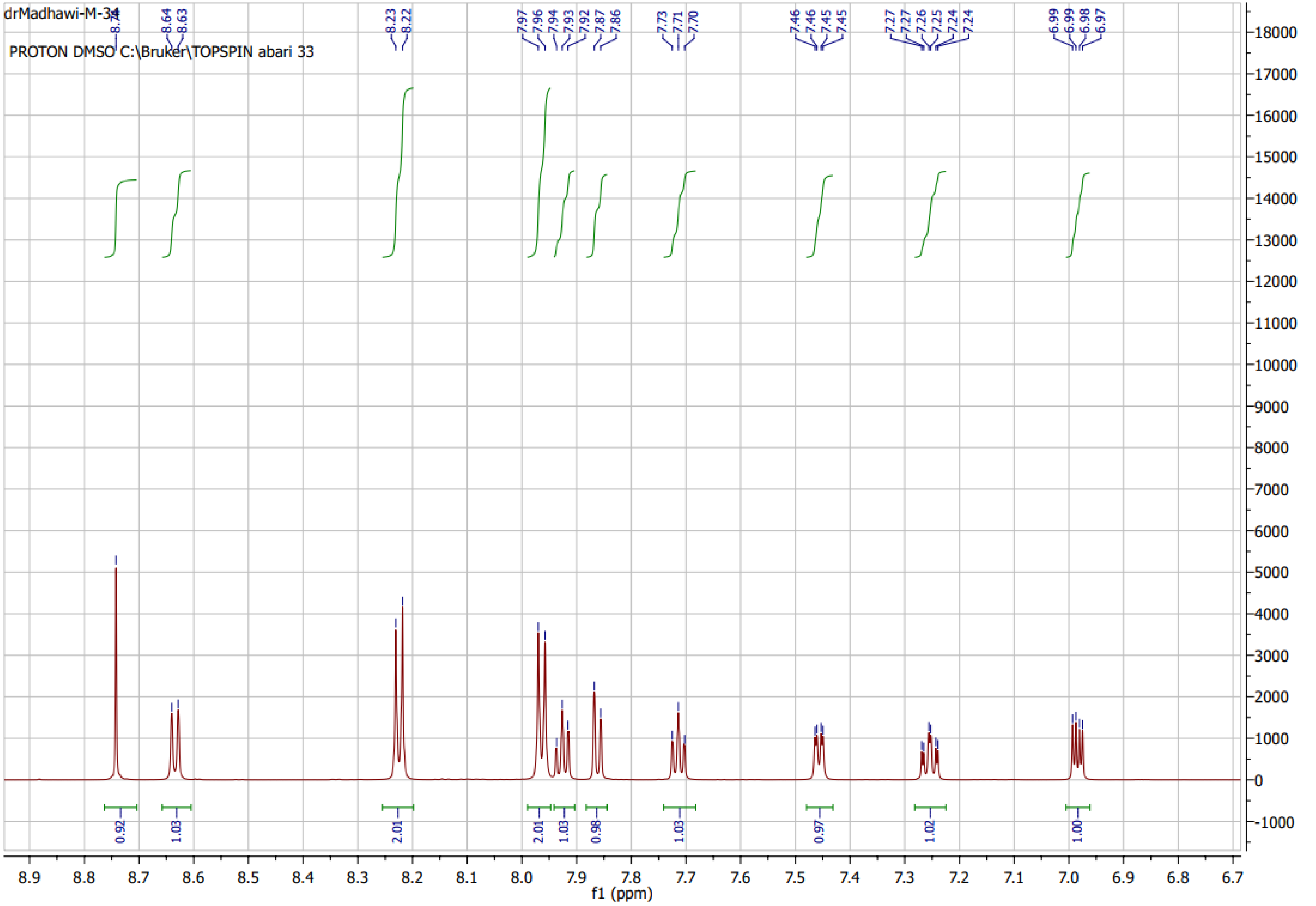

drMadhawi-M-34
PROTON DMSO C:\Bruker\TOPSPIN abari 33
8.74
8.64
8.63
8.23
8.22
7.97
7.96
7.94
7.93
7.92
7.87
7.86
7.73
7.71
7.70
7.46
7.46
7.45
7.45
7.27
7.27
7.26
7.25
7.24
7.24
6.99
6.99
6.98
6.97
0.92
1.03
2.01
2.01
1.03
0.98
1.03
0.97
1.02
1.00
f1 (ppm)

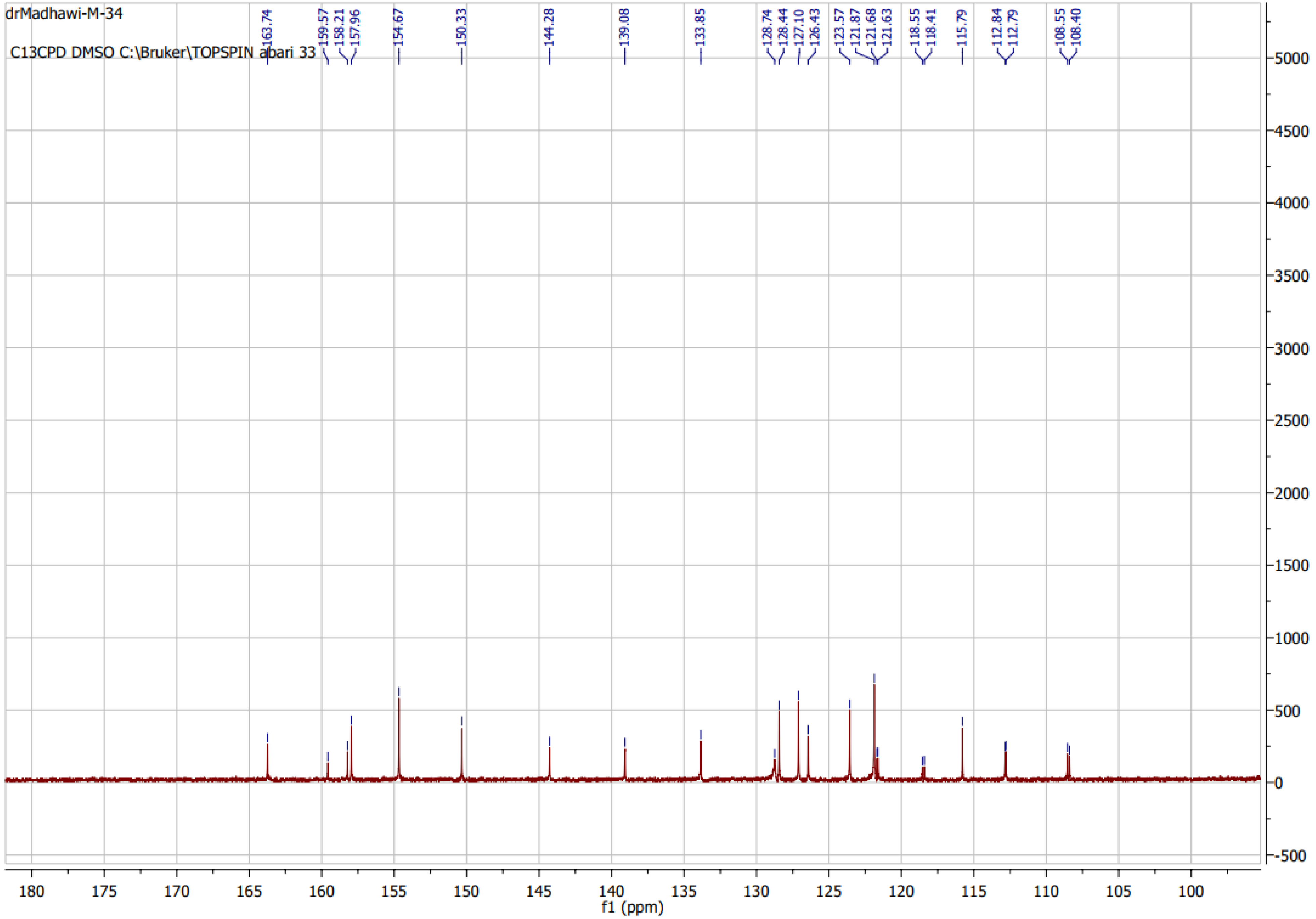

drMadhawi-M-34
C13CPD DMSO C:\Bruker\TOPSPIN abari 33
163.74
159.57
158.21
157.96
154.67
150.33
144.28
139.08
133.85
128.74
128.44
127.10
126.43
123.57
121.87
121.68
121.63
118.55
118.41
115.79
112.84
112.79
108.55
108.40
f1 (ppm)

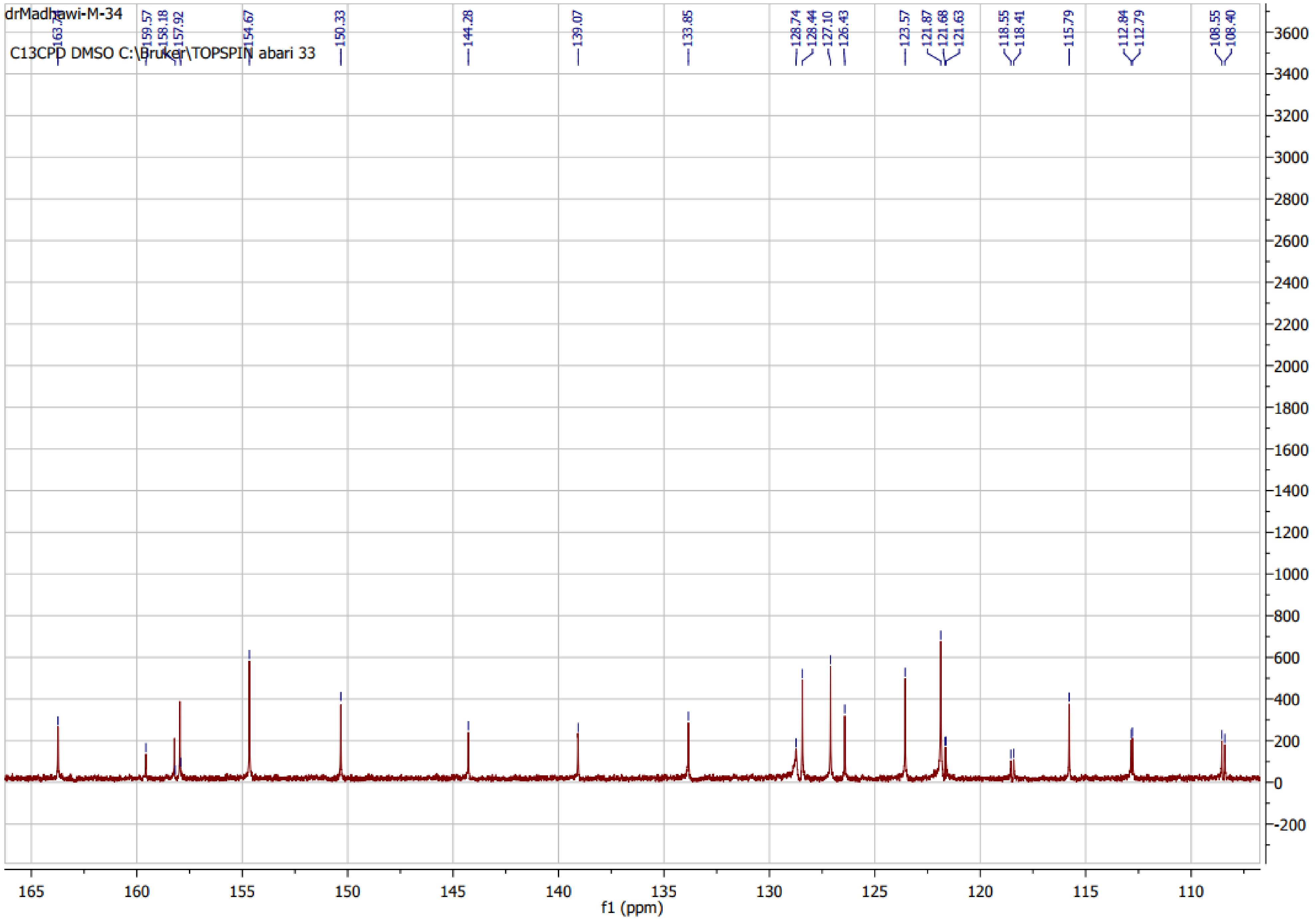
drMadhawi-M-34
C13CPD DMSO C:\Bruker\TOPSPIN abari 33
163.74
159.57
158.18
157.92
154.67
150.33
144.28
139.07
133.85
128.74
128.44
127.10
126.43
123.57
121.87
121.68
121.63
118.55
118.41
115.79
112.84
112.79
108.55
108.40
f1 (ppm)

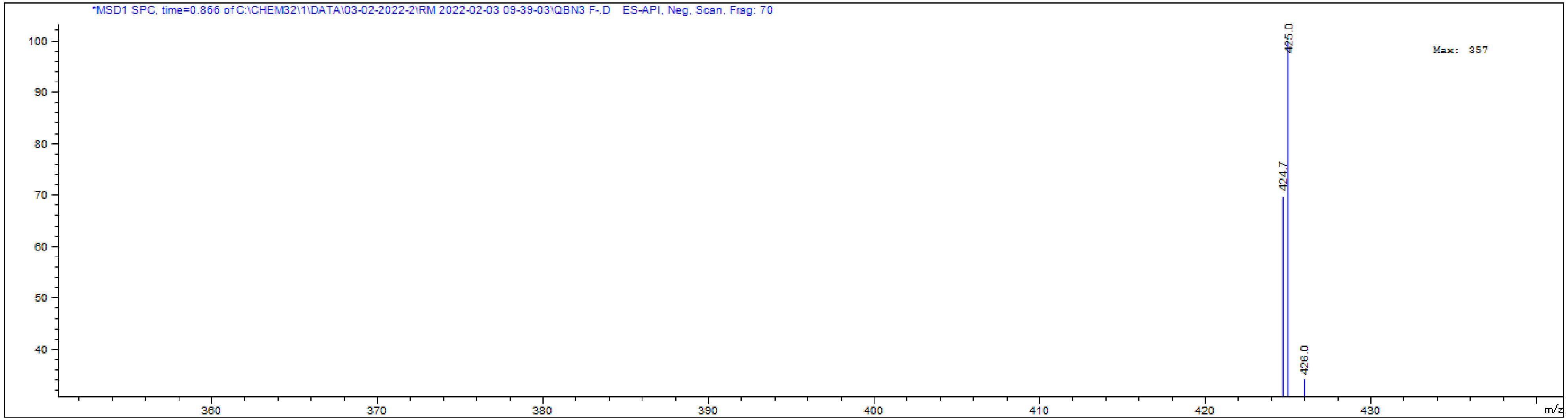

*MSD1 SPC, time=0.866 of C:\CHEM32\1\DATA\03-02-2022-2\RM 2022-02-03 09-39-03\QBN3 F-.D ES-API, Neg, Scan, Frag: 70
Max: 357
425.0
424.7
426.0
100
90
80
70
60
50
40
360
370
380
390
400
410
420
430
m/z

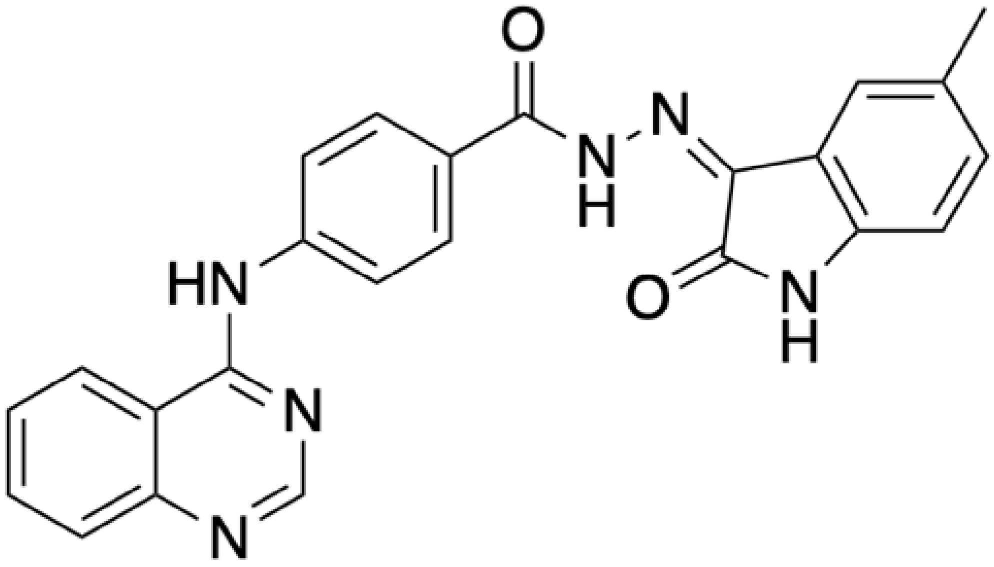

O
N
N
H
HN
O
N
H
N
N

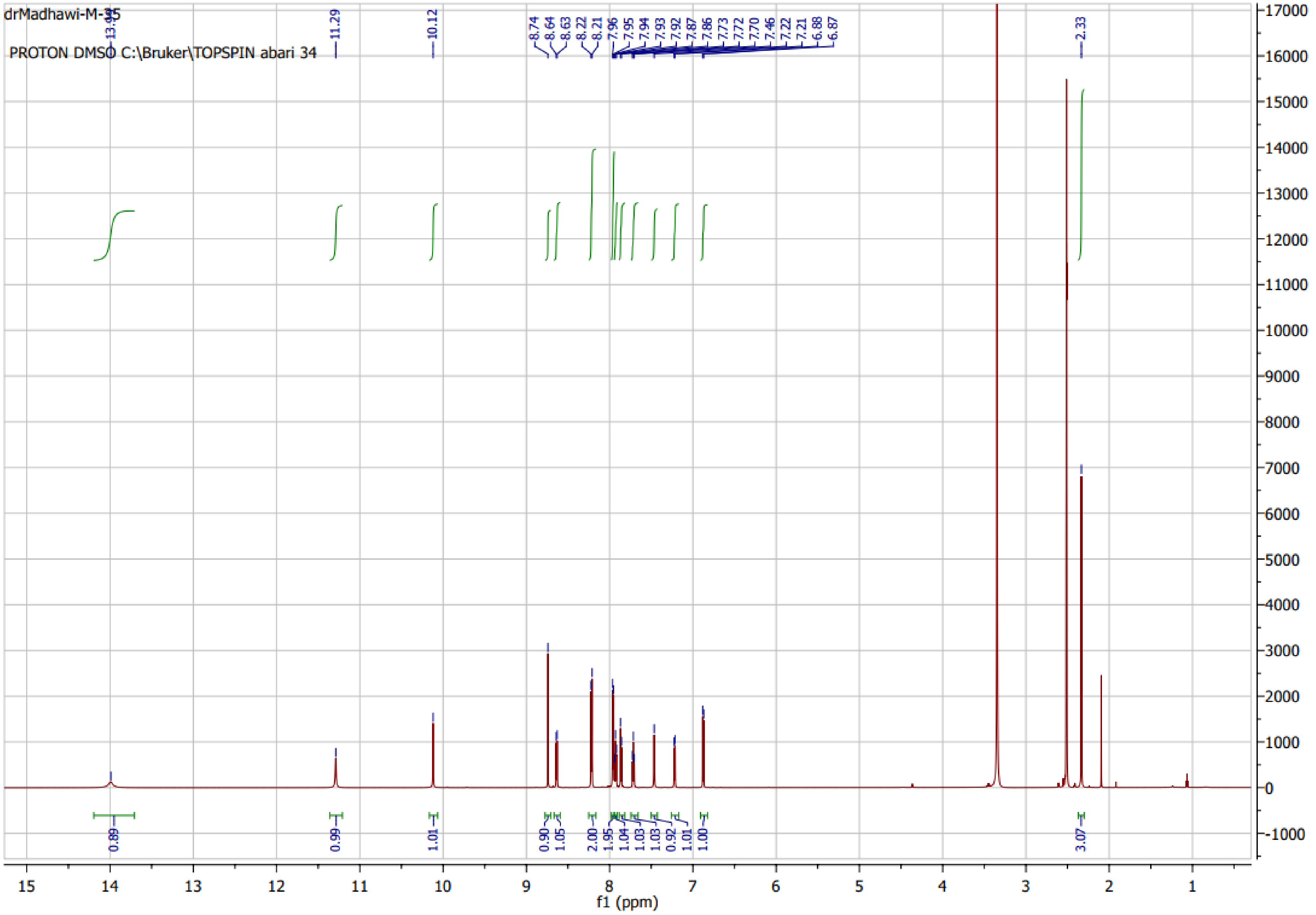

drMadhawi-M-35
PROTON DMSO C:\Bruker\TOPSPIN abari 34
13.99
11.29
10.12
8.74
8.64
8.63
8.22
8.21
7.96
7.95
7.94
7.93
7.92
7.87
7.86
7.73
7.72
7.70
7.46
7.22
7.21
6.88
6.87
2.33
0.89
0.99
1.01
0.90
1.05
2.00
1.95
1.04
1.03
1.03
0.92
1.01
1.00
3.07
f1 (ppm)

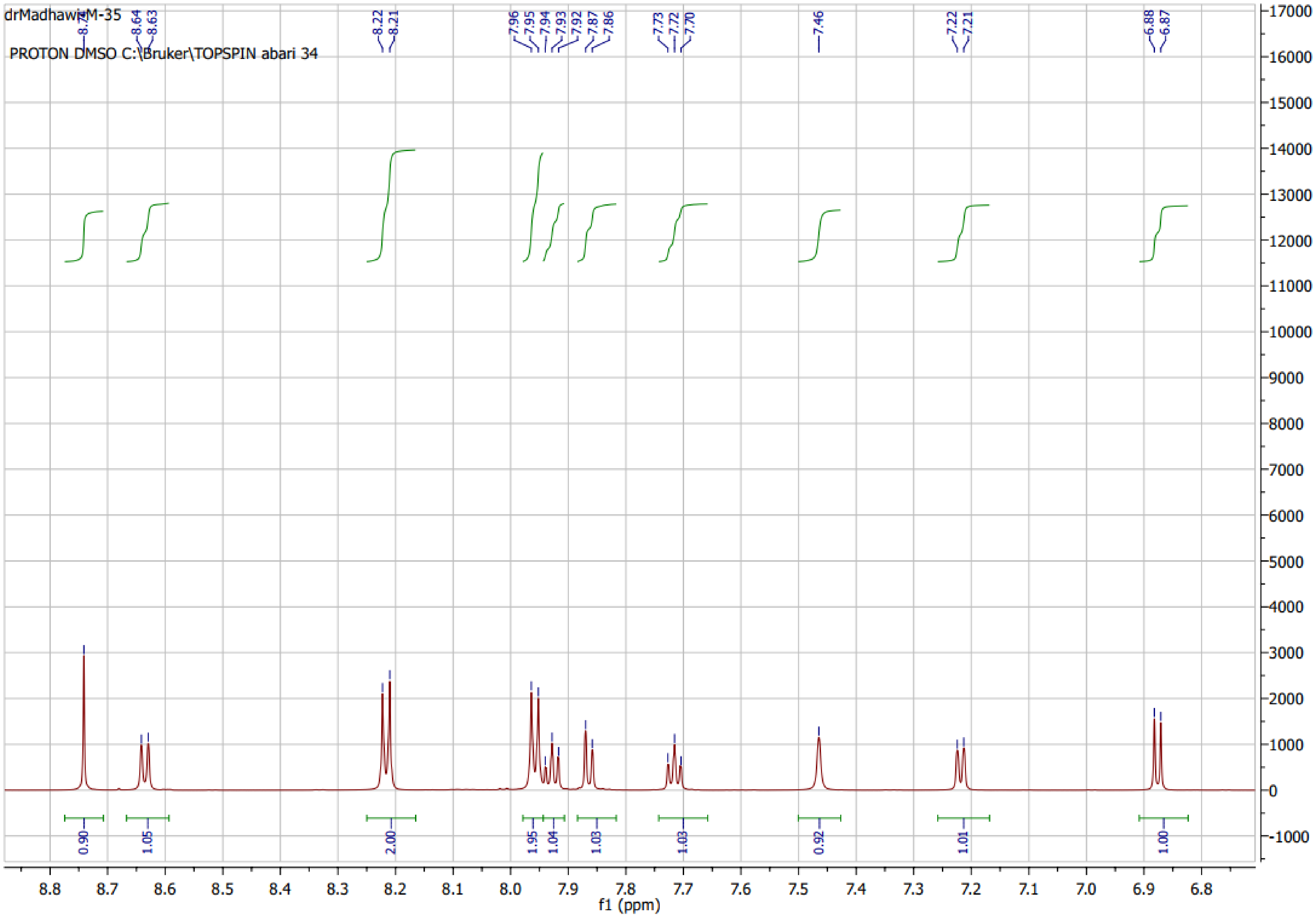
drMadhawiM-35
PROTON DMSO C:\Bruker\TOPSPIN abari 34
8.64
8.63
8.22
8.21
7.96
7.95
7.94
7.93
7.92
7.87
7.86
7.73
7.72
7.70
7.46
7.22
7.21
6.88
6.87
0.90
1.05
2.00
1.95
1.04
1.03
1.03
0.92
1.01
1.00
f1 (ppm)

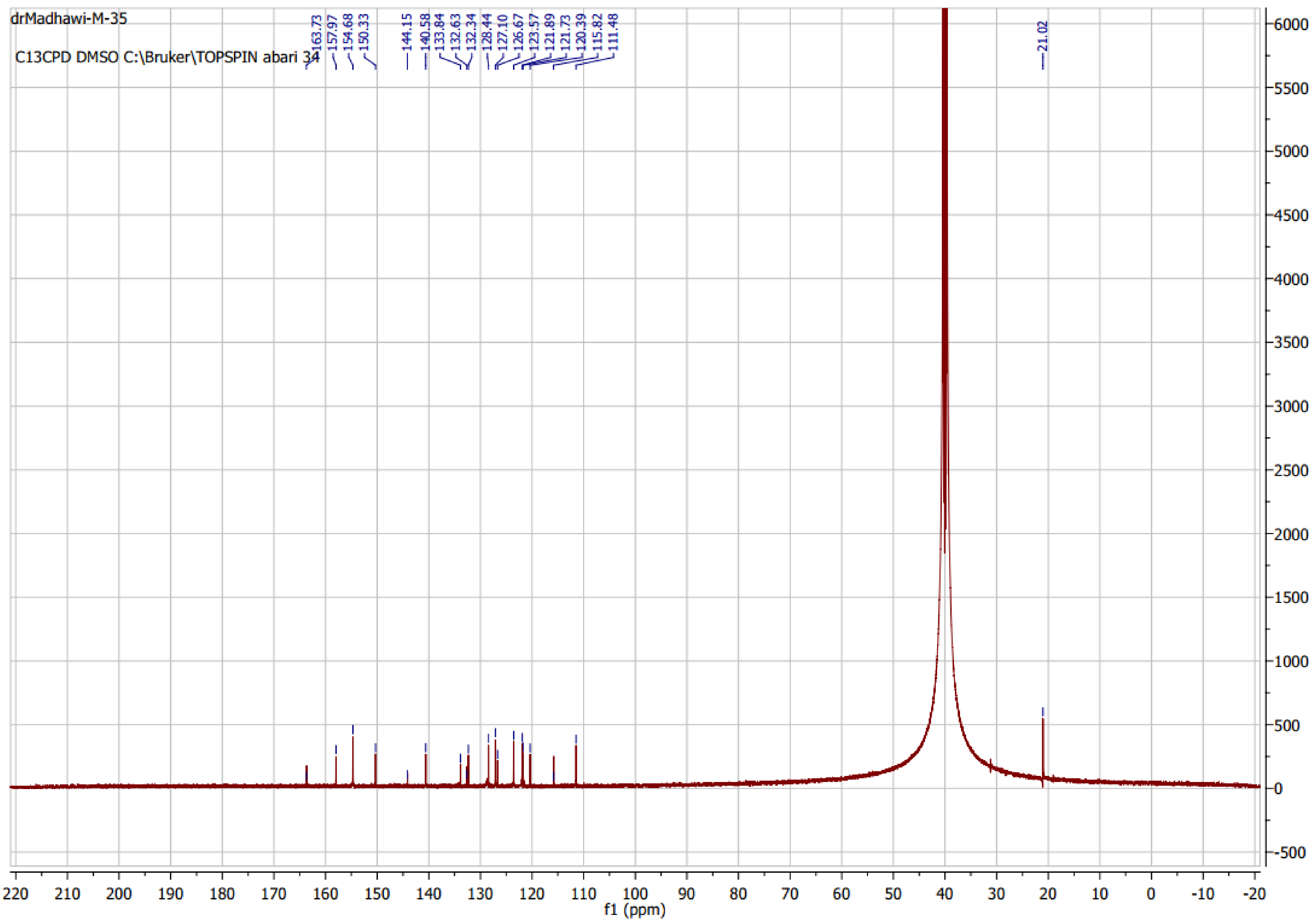

drMadhawi-M-35
C13CPD DMSO C:\Bruker\TOPSPIN abari 34
163.73
157.97
154.68
150.33
144.15
140.58
133.84
132.63
132.34
128.44
127.10
126.67
123.57
121.89
121.73
120.39
115.82
111.48
21.02
f1 (ppm)

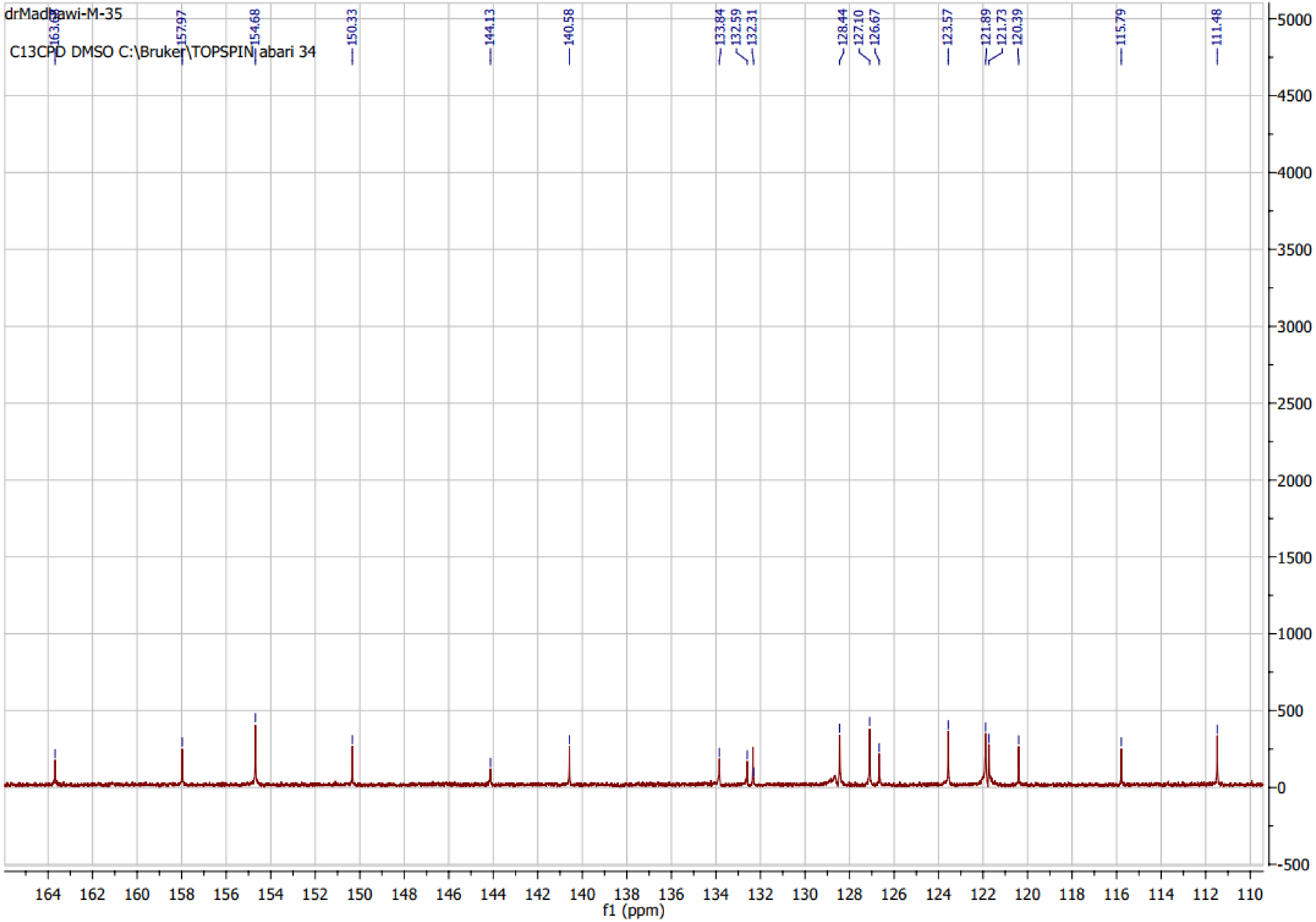
drMadbawi-M-35
C13CPD DMSO C:\Bruker\TOPSPIN abari 34
163.6
157.97
154.68
150.33
144.13
140.58
133.84
132.59
132.31
128.44
127.10
126.67
123.57
121.89
121.73
120.39
115.79
111.48
f1 (ppm)

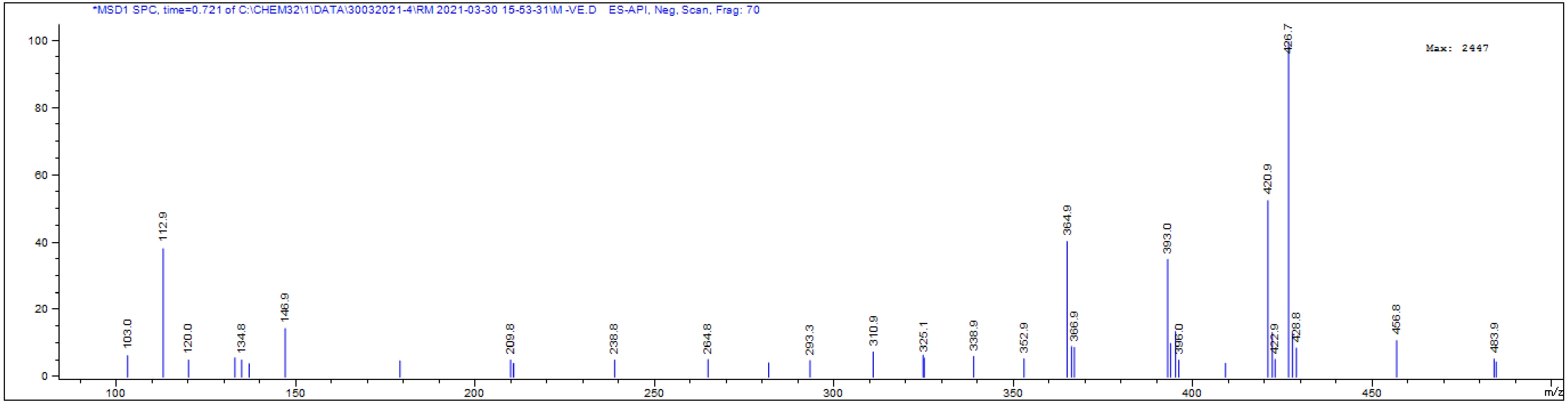
*MSD1 SPC, time=0.721 of C:\CHEM32\1\DATA\30032021-4\RM 2021-03-30 15-53-31\M -VE.D ES-API, Neg, Scan, Frag: 70
Max: 2447
103.0
112.9
120.0
134.8
146.9
209.8
238.8
264.8
293.3
310.9
325.1
338.9
352.9
364.9
366.9
393.0
395.0
420.9
422.9
426.7
428.8
456.8
483.9
m/z
